# Prognostic effect of whole chromosomal aberration signatures in standard-risk, non-WNT/non-SHH medulloblastoma: a retrospective, molecular analysis of the HIT-SIOP PNET 4 trial

**DOI:** 10.1016/S1470-2045(18)30532-1

**Published:** 2018-12

**Authors:** Tobias Goschzik, Edward C Schwalbe, Debbie Hicks, Amanda Smith, Anja zur Muehlen, Dominique Figarella-Branger, François Doz, Stefan Rutkowski, Birgitta Lannering, Torsten Pietsch, Steven C Clifford

**Affiliations:** a Department of Neuropathology, DGNN Brain Tumour Reference Center, University of Bonn Medical Center, Bonn, Germany; b Wolfson Childhood Cancer Research Centre, Northern Institute for Cancer Research, Newcastle University, Newcastle upon Tyne, UK; c Department of Applied Sciences, Northumbria University, Newcastle upon Tyne, UK; d Department of Pathology and Neuropathology, Assistance Publique Hôpitaux de Marseille, and Institute of Neurophysiopathology, UMR CNRS 7051 Aix Marseille University, Marseille, France; e SIREDO Cancer Center (Care, Innovation and Research in Pediatric, Adolescents and Young Adults Oncology), Institut Curie and University Paris Descartes, Paris, France; f University Medical Center Hamburg-Eppendorf, Hamburg, Germany; g Department of Pediatrics, University of Gothenburg and the Queen Silvia Children's Hospital, Gothenburg, Sweden

## Abstract

**Background:**

Most children with medulloblastoma fall within the standard-risk clinical disease group defined by absence of high-risk features (metastatic disease, large-cell/anaplastic histology, and *MYC* amplification), which includes 50–60% of patients and has a 5-year event-free survival of 75–85%. Within standard-risk medulloblastoma, patients in the WNT subgroup are established as having a favourable prognosis; however, outcome prediction for the remaining majority of patients is imprecise. We sought to identify novel prognostic biomarkers to enable improved risk-adapted therapies.

**Methods:**

The HIT-SIOP PNET 4 trial recruited 338 patients aged 4–21 years with medulloblastoma between Jan 1, 2001, and Dec 31, 2006, in 120 treatment institutions in seven European countries to investigate hyperfractionated radiotherapy versus standard radiotherapy. In this retrospective analysis, we assessed the remaining tumour samples from patients in the HIT-SIOP PNET 4 trial (n=136). We assessed the clinical behaviour of the molecularly defined WNT and SHH subgroups, and identified novel independent prognostic markers and models for standard-risk patients with non-WNT/non-SHH disease. Because of the scarcity and low quality of available genomic material, we used a mass spectrometry-minimal methylation classifier assay (MS-MIMIC) to assess methylation subgroup and a molecular inversion probe array to detect genome-wide copy number aberrations. Prognostic biomarkers and models identified were validated in an independent, demographically matched cohort (n=70) of medulloblastoma patients with non-WNT/non-SHH standard-risk disease treated with conventional therapies (maximal surgical resection followed by adjuvant craniospinal irradiation [all patients] and chemotherapy [65 of 70 patients], at UK Children's Cancer and Leukaemia Group and European Society for Paediatric Oncology (SIOPE) associated treatment centres between 1990 and 2014. These samples were analysed by Illumina 450k DNA methylation microarray. HIT-SIOP PNET 4 is registered with ClinicalTrials.gov, number NCT01351870.

**Findings:**

We analysed methylation subgroup, genome-wide copy number aberrations, and mutational features in 136 assessable tumour samples from the HIT-SIOP PNET 4 cohort, representing 40% of the 338 patients in the trial cohort. This cohort of 136 samples consisted of 28 (21%) classified as WNT, 17 (13%) as SHH, and 91 (67%) as non-WNT/non-SHH (we considered Group3 and Group4 medulloblastoma together in our analysis because of their similar molecular and clinical features). Favourable outcomes for WNT tumours were confirmed in patients younger than 16 years, and all relapse events in SHH (four [24%] of 17) occurred in patients with TP53 mutation (*TP53*^mut^) or chromosome 17p loss. A novel whole chromosomal aberration signature associated with increased ploidy and multiple non-random whole chromosomal aberrations was identified in 38 (42%) of the 91 samples from patients with non-WNT/non-SHH medulloblastoma in the HIT-SIOP PNET 4 cohort. Biomarkers associated with this whole chromosomal aberration signature (at least two of chromosome 7 gain, chromosome 8 loss, and chromosome 11 loss) predicted favourable prognosis. Patients with non-WNT/non-SHH medulloblastoma could be reclassified by these markers as having favourable-risk or high-risk disease. In patients in the HIT-SIOP PNET4 cohort with non-WNT/non-SHH medulloblastoma, with a median follow-up of 6·7 years (IQR 5·8–8·2), 5-year event-free survival was 100% in the favourable-risk group and 68% (95% CI 57·5–82·7; p=0·00014) in the high-risk group. In the validation cohort, with a median follow-up of 5·6 years (IQR 3·1–8·1), 5-year event-free survival was 94·7% (95% CI 85·2–100) in the favourable-risk group and 58·6% (95% CI 45·1–76·1) in the high-risk group (hazard ratio 9·41, 95% CI 1·25–70·57; p=0·029). Our comprehensive molecular investigation identified subgroup-specific risk models which allowed 69 (51%) of 134 accessible patients from the standard-risk medulloblastoma HIT-SIOP PNET 4 cohort to be assigned to a favourable-risk group.

**Interpretation:**

We define a whole chromosomal signature that allows the assignment of non-WNT/non-SHH medulloblastoma patients normally classified as standard-risk into favourable-risk and high-risk categories. In addition to patients younger than 16 years with WNT tumours, patients with non-WNT/non-SHH tumours with our defined whole chromosomal aberration signature and patients with SHH-*TP53*^wild-type^ tumours should be considered for therapy de-escalation in future biomarker-driven, risk-adapted clinical trials. The remaining subgroups of patients with high-risk medulloblastoma might benefit from more intensive therapies.

**Funding:**

Cancer Research UK, Swedish Childhood Cancer Foundation, French Ministry of Health/French National Cancer Institute, and the German Children's Cancer Foundation.

## Introduction

Medulloblastoma, the most common malignant childhood brain tumour, is now recognised as an umbrella term for different molecular pathological disease entities. These entities differ in their progenitor cells, characteristic mutations, biological profiles, and clinical behaviour. Currently, WHO classification of CNS tumours recognises four distinct genetically defined entities (WNT, SHH-*TP53*^wild-type^, SHH-*TP53*^mut^, and non-WNT/non-SHH).^1^ Non-WNT/non-SHH medulloblastoma encompasses Group3 and Group4, which were defined by epigenetic and mRNA expression signatures^2^ and are considered provisional variants by the 2016 WHO classification.^1^ Understanding the molecular pathology and clinical relevance of medulloblastoma subtypes provides substantial opportunities for personalised risk-adapted therapies.

**Research in context**

**Evidence before this study**

International consensus and the 2016 WHO classification recognise the following distinct clinico-molecular disease entities in medulloblastoma: WNT, SHH-*TP53*^wild-type^, SHH-*TP53*^mut^, and non-WNT/non-SHH (encompassing Group3 and Group4). Standard-risk, non-infant disease (with 75–85% 5-year progression-free survival and affecting 50–60% of patients) represents the largest clinical treatment group of patients. The ongoing pan-European SIOP PNET 5 MB clinical trial defines standard-risk, non-infant disease as the absence of high-risk clinical features such as metastatic disease or subtotal resection, molecular features (*MYC* or *MYCN* amplification or *TP53* mutation in SHH medulloblastoma), and histological characteristics (large-cell/anaplastic disease). These definitions were established based on previous disease-wide studies. The SIOP PNET 5 MB trial is investigating reduced-intensity therapies for patients classified as standard-risk with expected good prognosis (ie, WNT medulloblastoma), aimed at maintaining overall survival while minimising late toxicities. However, biomarkers that stratify risk within remaining standard-risk patients with non-WNT medulloblastoma have not been identified. Moreover, novel non-WNT/non-SHH medulloblastoma epigenetic subtypes have been recognised; however, these subtypes remain to be validated and implemented clinically. Our own reviews of the literature formed the foundation for the present study; we did not carry out any formal literature searches before the study start date (December, 2015).

**Added value of this study**

To our knowledge, HIT-SIOP PNET 4 is the only completed pan-European clinical trial in patients with standard-risk medulloblastoma. However, to date, systematically collected biological material remaining from this trial was not amenable to contemporary molecular analysis. Application of novel methods to enable assessment of this cohort, and investigation of an independent demographically matched standard-risk medulloblastoma validation cohort, allowed derivation and validation of biomarker-driven, risk-stratification models on the basis of the molecular pathology of standard-risk medulloblastoma, including a novel whole chromosomal cytogenetic aberration signature within standard-risk non-WNT/non-SHH medulloblastoma. These newly described whole chromosomal cytogenetic aberration signatures allowed reallocation of more than 50% of HIT-SIOP PNET 4 patients with standard-risk medulloblastoma into a favourable-risk group, while the remaining patients were classified as high risk. Therefore, findings from this study resolve current patients with standard-risk medulloblastoma into biomarker-defined distinct favourable-risk and high-risk groups, and represent a substantial step in our ability to risk stratify and clinically manage medulloblastoma.

**Implications of all the available evidence**

The results of this study redefine the concepts of risk stratification in standard-risk medulloblastoma, providing insight into its molecular subtypes, their underpinning biology, and clinical application. Stratification of standard-risk medulloblastoma by use of the biomarkers and validated schemes we describe could allow assignment of 150–200 patients per year in Europe into a favourable-risk group, and such patients could benefit from reduction of treatment intensity. Patients not classified as favourable-risk should be considered high-risk and might benefit from treatment intensification. The molecular risk groups and biomarker schemes presented in this study are amenable to routine diagnostic assessment and provide a foundation for future clinical trials and research investigations.

Discovery and validation of clinically meaningful medulloblastoma features in previous clinical trial cohorts have driven advances in the clinical management of the disease. Children younger than 16 years of age at diagnosis with WNT-activated medulloblastomas have consistently achieved favourable outcomes (5-year event-free survival >95%),^3^, ^4^ whereas other disease features, including *MYC* or *MYCN* amplification, large-cell/anaplastic histology, metastatic disease, or subtotal resection, define high-risk disease (5-year event-free survival <60%).^5^ These disease features now underpin risk-adapted therapies in ongoing biomarker-driven international prospective clinical studies, such as the SIOP PNET 5 MB (NCT02066220) and SJMB12 (NCT01878617) trials, which aim to improve outcomes through reduced-intensity therapies for favourable-risk patients and randomised assessment of adapted therapies in the remaining patients.

Standard-risk medulloblastoma represents the predominant clinical treatment group (around 60% of patients) and is defined by the absence of clinical, molecular, and histopathological high-risk features. This group encompasses tumours of all variants except high-risk SHH-*TP53*^mut^.^6^, ^7^ Diagnosis of favourable-risk, WNT disease (around 20% of patients with standard-risk medulloblastoma) provides a clear precedent for therapy de-escalation within clinical trials. By contrast, patients with non-WNT, standard-risk medulloblastoma have heterogeneous outcomes (5-year event-free survival around 75%), and further actionable risk groups are yet to be identified or validated to the point of clinical application. The favourable risk of patients with standard-risk, SHH-*TP53*^wild-type^ medulloblastoma^6^, ^7^ identified in retrospective series requires validation in clinical trials, and reproducible and clinically significant molecular pathological features within non-WNT/non-SHH tumours remain to be defined. Research has found that Group3 and Group4 medulloblastomas represent heterogeneous, biologically overlapping entities—few recurrent mutations have been observed, whole chromosomal cytogenetic aberrations are common,^8^, ^9^, ^10^, ^11^ and evidence of novel molecular subtypes is emerging.^6^, ^12^, ^13^

To our knowledge, HIT-SIOP PNET 4^14^ is the first completed, international, prospective clinical trial of non-metastatic childhood medulloblastoma (patients aged 4–21 years at diagnosis) and this cohort of patients represents a unique opportunity to explore the molecular pathology of standard-risk medulloblastoma, its potential for risk stratification, and the development of new therapeutic concepts. Trial participants were postoperatively staged and randomly assigned to treatment with standard or hyperfractionated radiotherapy, followed by chemotherapy with eight cycles of cisplatin, lomustine, and vincristine. No difference in event-free survival was observed between the two treatment groups.^14^

Formalin-fixed, paraffin-embedded (FFPE) tumour material for biological studies was prospectively collected, which enabled confirmation of favourable outcomes in patients with WNT medulloblastoma (defined by immunohistochemistry [IHC]) and identification of chromosome 17 imbalances on a diploid background (17p loss and/or 17q gain, by fluorescence in-situ hybridisation [FISH]) as a marker of poor prognosis.^15^ However, until now, contemporary molecular characterisation of the HIT-SIOP PNET 4 cohort, and assessment of its clinical relevance, has been restricted by the low quality and quantity of remaining tumour material.

In this Article, we report comprehensive molecular and pathological characterisation of the HIT-SIOP PNET 4 cohort using novel technologies^16^, ^17^ developed and adapted for assessment of the remnant tumour material. This analysis, alongside an independent, demographically matched, standard-risk medulloblastoma validation cohort, enabled the discovery and validation of concerted whole chromosomal aberration signatures with prognostic value for patients with non-WNT/non-SHH medulloblastoma. We describe the development of risk stratification models for standard-risk, non-WNT/non-SHH disease, which might allow reassignment of all patients with standard-risk medulloblastoma into biomarker-defined favourable-risk or high-risk groups.

## Methods

### Study design and participants

In this retrospective analysis, we assessed remaining tumour samples from patients from the HIT-SIOP PNET 4 trial (NCT01351870).^14^ Between Jan 1, 2001, and Dec 31, 2006, 338 patients were recruited from 120 different treatment centres in seven European countries (France, Germany, Italy, the Netherlands, Spain, Sweden, and the UK; appendix p 3). The study investigated treatment outcomes in patients aged 4–21 years using either hyperfractionated radiotherapy or standard delivery radiotherapy followed by chemotherapy.^1^ Standard delivery radiotherapy comprised 23·4 Gy to the craniospinal axis and 54 Gy to the whole posterior fossa, and was given over 42 days in 30 fractions of 1·8 Gy each day for 5 days per week. Hyperfractionated radiotherapy was given in 68 fractions at 1·0 Gy twice per day with an 8 h interval between fractions, given over 48 days. The total craniospinal dose was 36 Gy, and the whole posterior fossa dose was 60 Gy, with a further boost to 68 Gy to the tumour bed. Adjuvant chemotherapy was started 6 weeks after the end of radiotherapy. Eight cycles of cisplatin (70 mg/m^2^ intravenously) and lomustine (75 mg/m^2^) on day 1, and vincristine (1·5 mg/m^2^ intravenously) on days 1, 8, and 15, were given with a 6 week interval between each cycle.^14^

Minute remnant material (cytospin-concentrated cellular nuclei preparations) or tumour sections, originally intended for FISH and IHC,^15^ were available for analysis (samples from 147 patients). We retained tumours from patients with subtotally resected disease^18^ or categorised as *MYCN*-amplified to assess their prognostic value in a clinically controlled cohort.^6^, ^11^, ^15^ We excluded *MYC*-amplified tumours because of their established poor prognosis.^5^ 136 tumour samples met these criteria and underwent molecular investigation. The demographics of the patients who provided these tumour samples (clinical and molecular cohort) and their prognostic features were consistent with the whole trial cohort (table).

**Table:** Clinical and molecular characteristics of all cohorts

		**Clinical cohort**	**Clinical and molecular standard-risk cohort**		
		All patients in HIT-SIOP PNET 4 (n=338)	All subgroups in HIT-SIOP PNET 4 (n=136)	Non-WNT/non-SHH in HIT-SIOP PNET 4 (n=91)	Non-WNT/non-SHH in validation cohort (Newcastle; n=70)
Sex					
	Male	211 (62%)	81 (60%)	61 (67%)	50 (71%)
	Female	127 (38%)	55 (40%)	30 (33%)	20 (29%)
	Male:female ratio	1·66:1	1·5:1	2:1	2·5:1
Age at diagnosis (years)*		9·0 (3–20) [7·0–12·0]	9·0 (3–20) [7·0–12·0]	8·0 (4–20) [6·0–10·0]	8·5 (4–18) [8·8–11·4]
Treatment					
	Standard radiotherapy	169 (50%)	67 (49%)	43 (47%)	66 (94%)
	Hyperfractionated radiotherapy	169 (50%)	69 (51%)	48 (53%)	4 (6%)
Histology					
	Classic	273 (81%)	111 (82%)	81 (89%)	64 (91%)
	Desmoplastic/nodular	47 (14%)	25 (18%)	10 (11%)	6 (9%)
	Large-cell/anaplastic	16 (5%)†	0	0	0
	No review	2 (1%)	0	0	0
Resection					
	Gross total resection	286 (90%)	121 (92%)	81 (92%)	54 (80%)
	Subtotal resection	31 (10%)	10 (8%)	7 (8%)	14 (20%)
Follow-up (years)		6·6 (5·6–8·5)	6·7 (5·6–8·4)	6·7 (5·8–8·2)	5·6 (3·1–8·1)
Collection era (years)		2001–06	2001–06	2001–06	1990–2014‡
Molecular subgroup					
	WNT	··	28 (21%)	0	0
	SHH	··	17 (13%)	0	0
	Group3	··	15 (11%)	15 (16%)	6 (9%)
	Group4	··	76 (56%)	76 (84%)	64 (91%)
β-catenin immunohistochemistry					
	Total assessed	··	121	56	28
	Nuclear accumulation	··	30 (25%)	0	1 (4%)
	Normal	··	91 (75%)	56 (100%)	27 (96%)
*CTNNB1* mutation					
	Total assessed	··	114	51	56
	Mutant	··	26 (23%)	0	0
	Wild-type	··	88 (77%)	51 (100%)	56 (100%)
*TP53* mutation in SHH					
	Total assessed	··	15	0	0
	SHH-*TP53*^wild-type^	··	11 (73%)	0	0
	SHH-*TP53*^mut^	··	4 (27%)	0	0
*MYC* amplification					
	Amplified	··	0	0	0
	Not amplified	··	136 (100%)	91 (100%)	70 (100%)
*MYCN* amplification					
	Amplified	··	10 (7%)	10 (11%)	6 (9%)
	Not amplified	··	126 (93%)	81 (89%)	64 (91%)
Chromosome 17 (interphase fluorescence in-situ hybridisation)					
	Total assessed	··	101	69	
	17p loss or 17q gain (diploid(cen))	··	17 (17%)	15 (22%)	NA
	Others	··	84 (83%)	54 (78%)	NA

Data are n (%), median (IQR) or n, unless otherwise indicated. Some percentages do not total 100 because of non-assessable tumours. NA=not analysed.

* Data are median (range) [IQR].

† The trial was amended in 2003 to exclude cases with large-cell/anaplastic histology.

‡ Median year of diagnosis 2006.

We validated and extended our findings in a second independent, demographically matched, retrospective cohort of patients with non-WNT/non-SHH standard-risk medulloblastoma (n=70) collected at UK Children's Cancer and Leukaemia Group and European Society for Paediatric Oncology (SIOPE) associated treatment centres between 1990 and 2014. Patients in this cohort received equivalent therapies (maximal surgical resection [all patients], adjuvant craniospinal radiotherapy [all patients; standard radiotherapy in variable doses—low dose: 24–27 Gy, 39 patients; high dose: 35–39 Gy, 27 patients; hyperfractionated radiotherapy variable doses: 32·4 Gy craniospinal radiotherapy plus 23·4 Gy boost, one patient; 60 Gy hyperfractionated accelerated radiotherapy, one patient; 31/59 Gy, one patient; and 39/54, one patient], and chemotherapy [65 (93%) of 70 patients]).

Written informed consent for tumour collection for biological studies was obtained from patients or their parents. Tumour investigations were done with approval from Newcastle and North Tyneside Research Ethics Committee (study reference 07/Q0905/71)—all tumour material was collected in accordance with this approval.

### Procedures

Because only material of mostly low quantity and quality was available, the HIT-SIOP PNET 4 samples were unsuitable for subgroup assessment using conventional approaches (DNA methylation array^19^ or mRNA expression analysis by Nanostring^20^); therefore, we analysed all samples using a mass spectrometry-minimal methylation classifier (MS-MIMIC) assay to assess their molecular subgroup.^16^ For the validation cohort, samples were of sufficient quality and quantity to do Illumina 450k DNA methylation microarray (62 DNA samples were from frozen material and eight were from FFPE tissue) and consensus methylation subgroup was assigned as described previously.^6^

We assessed amplification of *MYC* and *MYCN* oncogenes by interphase FISH^15^ and estimated gene copy numbers from molecular inversion probe and DNA methylation arrays,^19^ as previously described. We analysed mutations in exons 4–9 of *TP53* and exon 3 of *CTNNB1* with Sanger sequencing as previously described.^21^ We assessed mutations in *APC* using a customised next-generation DNA sequencing panel (Illumina; San Diego, CA, USA) in samples with *CTNNB1*^wild-type^ WNT medulloblastoma. We used a molecular inversion probe array (335 000 inversion probes; version 2.0; Affymetrix; Santa Clara, CA, USA) to identify aberrant changes in genomic copy number in samples from the HIT-SIOP PNET 4 trial.^17^ Raw molecular inversion probe data were analysed using Nexus Copy Number 7.0 Discovery Edition (BioDiscovery; El Segundo, CA, USA). We used SNP-FASST2 segmentation algorithm to make copy number and loss of heterozygosity estimations. We used GISTIC (Genomic Identification of Significant Targets in Cancer, v 1.0) to identify focal chromosomal aberrations (appendix pp 10–12).^22^ We analysed the validation cohort samples on the Illumina 450k DNA methylation microarray (Illumina; San Diego, CA, USA), and estimated chromosomal and focal copy number changes by use of the R package conumee v 1.13.0, as previously described.^6^ We defined a whole chromosomal aberration group of patients by hierarchical clustering of recurrent (ie, >15%) aberrations.

Event-free survival was defined as the time from surgery to first event (progression or relapse), or date of last follow-up. Patients whose follow-up time exceeded 10 years were right-censored at 10 years. Clinical follow-up data were collected according to the HIT-SIOP PNET 4 trial protocol.^14^ For the validation cohort, clinical data were collected in the same format from individual treatment centres.

### Statistical analysis

Using hierarchical clustering, we clustered samples classified as non-WNT/non-SHH medulloblastoma subtype by their recurrent whole chromosomal aberration (ie, incidence >15%; appendix p 2). After molecular subgrouping, we observed similar cytogenetic changes and event-free survival between the non-WNT/non-SHH medulloblastomas subclassified as Group3 and Group4 (appendix p 9). Because of these results and the emerging evidence of their shared biology,^6^, ^12^ we considered these groups together in subsequent event-free survival analyses.

To test the null hypothesis that event-free survival was not associated with clinical, molecular, or pathological variables in patients with Group3 or Group4 medulloblastoma, we constructed Kaplan-Meier curves and compared patient groups with log-rank tests.

Using Cox modelling, we tested the prognostic value of clinical markers (gender, radiotherapy type [hyperfractionated *vs* standard], resection outcome [subtotal *vs* fully-resected disease], *MYCN* amplification [yes *vs* no], histology type [desmoplastic/nodular *vs* classic histology]), cytogenetic markers (recurrent whole chromosomal aberration [presence *vs* absence]), and cumulative numbers of total whole chromosomal aberrations (gains *vs* losses). We verified the proportionality assumption for Cox modelling using scaled Schoenfeld residuals. We derived pragmatic assignments of patient risk by combining whole chromosomal aberrations that were significantly different in univariate testing to define risk groups and assessed their predictive value by calculating total area under the curve (AUC), sensitivity, and specificity at 5 years since diagnosis (appendix p 2). We clustered the tumour samples from the validation cohort by the recurrent whole chromosomal aberrations previously identified in the HIT-SIOP PNET 4 trial standard-risk, non-WNT/non-SHH medulloblastoma cohort and validated the derived risk stratification schemes.

Finally, to better understand the nature of the identified risk groups, we classified the validation cohort according to the recently published refinements of epigenetically defined substructures within non-WNT/non-SHH medulloblastoma.^6^, ^12^ Validation cohort samples were assigned to subgroup variants according to these published studies and visualised using *t*-distributed stochastic neighbour embedding (appendix pp 2–3).

We set the significance threshold at p<0·05 for all statistical tests in this study, and two-tailed p values are reported. We assessed significance of association using Fisher's exact test, and visualised the strength of associations using χ^2^ test residuals.

Further detailed methods are provided in the appendix pp 1–3. Statistical and bioinformatic analyses were done with R (version 3.4.2).

### Role of the funding source

The funders of the study had no role in study design, data collection, data analysis, data interpretation, or writing of the report. The corresponding author had full access to all the data in the study and had final responsibility for the decision to submit for publication.

## Results

**Figure 1:**
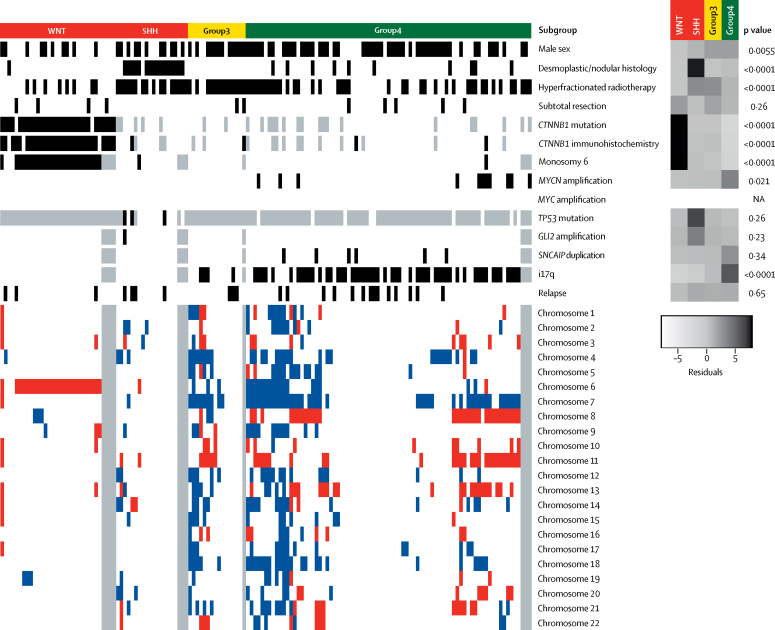
Clinical and disease-associated molecular features of the HIT-SIOP PNET 4 cohort All 147 patient samples available from the HIT-SIOP PNET 4 cohort with subgroup information are shown, including 11 samples without data on chromosomal aberrations. Black indicates positivity for an assessed feature (for sex, black indicates male and white indicates female). Grey indicates missing data. Red indicates chromosomal losses and blue indicates chromosomal gains. NA=not assessed. Residual scores from χ^2^ tests of association are shown (darker shades of grey indicate stronger enrichment) alongside p values from Fisher's exact tests.

We successfully assessed methylation subgroup (appendix p 4), genome-wide copy number aberrations (appendix p 4), and mutational features in 136 tumour samples from the HIT-SIOP PNET 4 cohort, recruited from 2001 to 2006 and representing 136 (40%) of 338 patients in the trial.^14^ Cohort clinical and molecular characteristics are summarised in figure 1 and the table.

Our integrative analysis found the expected distributions of clinical, pathological, and molecular features within WHO-defined medulloblastoma entities and their provisional sub-variants^1^ in the HIT-SIOP PNET 4 cohort (*CTNNB1* mutation and chromosome 6 monosomy in WNT medulloblastoma; desmoplastic/nodular pathology and *TP53* mutation in SHH medulloblastoma; i17q in non-WNT/non-SHH-medulloblastoma; and *SNCAIP* duplication and *MYCN* amplification in Group4; figure 1). Patients with WNT medulloblastoma (28 [21%] of 136) and patients with Group4 disease (76 [56%] of 136) were enriched in HIT-SIOP PNET 4 standard-risk medulloblastoma compared with retrospective disease-wide series,^8^, ^9^, ^10^, ^11^ as anticipated following exclusion of children younger than 4 years, adults older than 21 years, and patients with high-risk or metastatic disease from this cohort.

The prognostic relevance and demographic distribution of key clinical features across the study cohorts were compatible with our previous reports of the entire HIT-SIOP PNET 4 trial (table).^14^, ^15^

**Figure 2:**
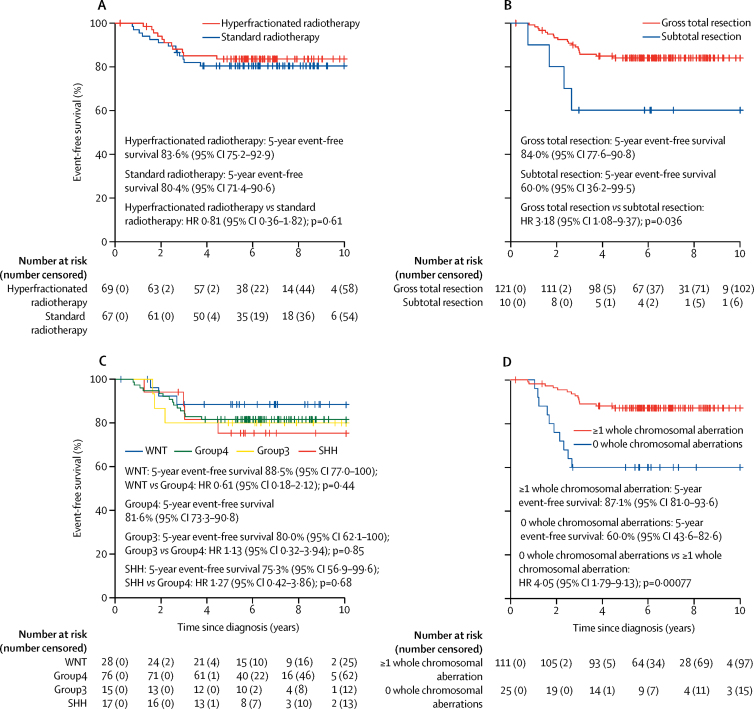

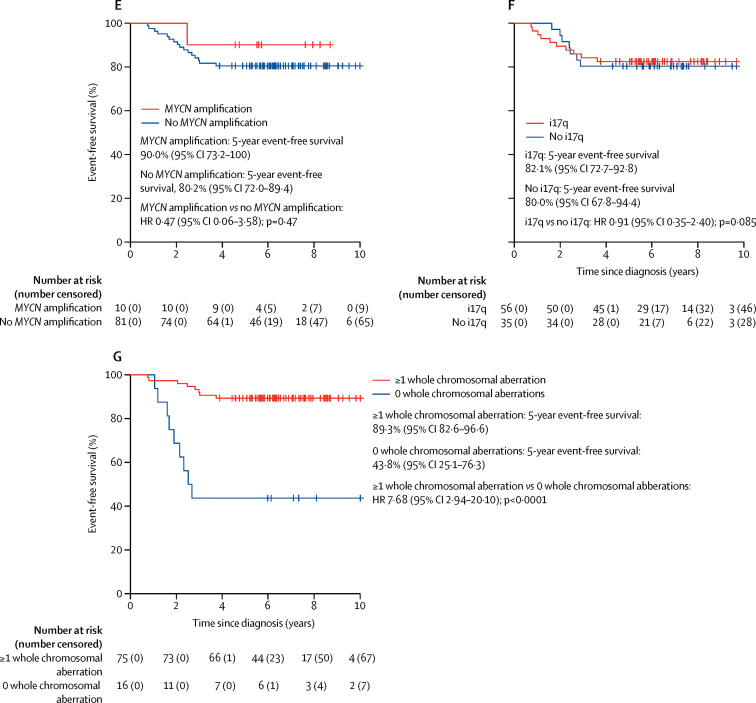
Event-free survival in the HIT-SIOP PNET 4 cohort by clinical and disease-associated molecular features Patients (n=136) were grouped as (A) treated with standard radiotherapy *vs* hyperfractionated radiotherapy, (B) those who had a gross total resection *vs* subtotal resection, (C) classified as per the four consensus medulloblastoma molecular subgroups, and (D) those with or without whole chromosomal aberrations. Event-free survival for patients with non-WNT/non-SHH disease (n=91) grouped as (E) patients with *MYCN* amplified *vs* non-amplified tumours, (F) patients with medulloblastomas presenting an i17q or not, and (G) patients with medulloblastomas with or without whole chromosomal aberration. HR=hazard ratio.

With a median follow-up of 6·7 years (IQR 5·6–8·4) in the HIT-SIOP PNET 4 cohort, 5-year event-free survival was equivalent between patients who received standard radiotherapy and those who received hyperfractionated radiotherapy (hazard ratio [HR] 0·81, 95% CI 0·36–1·82; p=0·61; figure 2), while patients who had a subtotal resection had a poorer event-free survival at 5 years than those who underwent gross total resection (HR 3·18, 1·08–9·37; p=0·036; figure 2). We found no differences in terms of 5-year event-free survival between the four methylation subgroups (figure 2; WNT 5-year event-free survival 88·5%, 95% CI 77·0–100; Group4 5-year event-free survival 81·6%, 73·3–90·8; Group3 5-year event-free survival 80·0, 62·1–100; SHH 5-year event-free survival 75·3%, 56·9–99·6; WNT *vs* Group4 HR 0·61, 95% CI 0·18–2·12, p=0·44; SHH *vs* Group4 1·27, 0·42–3·86, p=0·68; Group3 *vs* Group4 1·13, 0·32–3·94, p=0·85). Group3 and Group4 had very similar event-free survival curves (figure 2). By contrast, we found a significant association between the presence of whole chromosomal aberrations and favourable event-free survival outcomes compared with the absence of whole chromosomal aberrations (HR 4·05, 95% CI 1·79–9·13; p=0·00077; figure 2).

We investigated the clinical, molecular, and event-free survival characteristics of WHO-defined medulloblastoma molecular entities in the HIT-SIOP PNET 4 cohort.^1^ 25 (89%) of 28 WNT tumours showed the characteristic chromosome 6 monosomy and few other aberrations (appendix pp 5–6). We identified *CTNNB1* mutations in 26 (93%) of 28 WNT tumours (appendix pp 5–6). Both *CTNNB1* wild-type tumours showed a copy neutral loss of heterozygosity within chromosome 5q (*APC*) and we identified *APC* frameshift deletions (E1309fs ΔAAAAG and Q1062fs ΔACAAA). Outcomes within the WNT subgroup were age-dependent. We observed a 5-year event-free survival of 100% in patients younger than 16 years at diagnosis, and all WNT relapses (three [11%] of 28 WNT tumours) occurred in patients aged 16–20 years (p=0·00050; appendix pp 5–6).

Tumours classified as SHH in the HIT-SIOP PNET 4 cohort (17 [13%] of 136 patients) also had few whole chromosomal aberrations (appendix pp 7–8). Chromosome 17p loss (*TP53*) and *TP53* mutations were associated with each other (p=0·0090; appendix pp 7–8) and with worse event-free survival. All four (100%) of four events (relapses) were in patients with *TP53* mutation or chromosome 17p loss (p=0·0036). We did not observe *MYCN* amplifications in tumours classified as SHH medulloblastoma, including *TP53*^mut^ tumours. A previously reported SHH disease risk model (of chromosome 14 loss and *GLI2* amplification)^11^ showed significantly worse event-free survival for patients in this cohort (p=0·00067; appendix pp 7–8).

**Figure 3:**
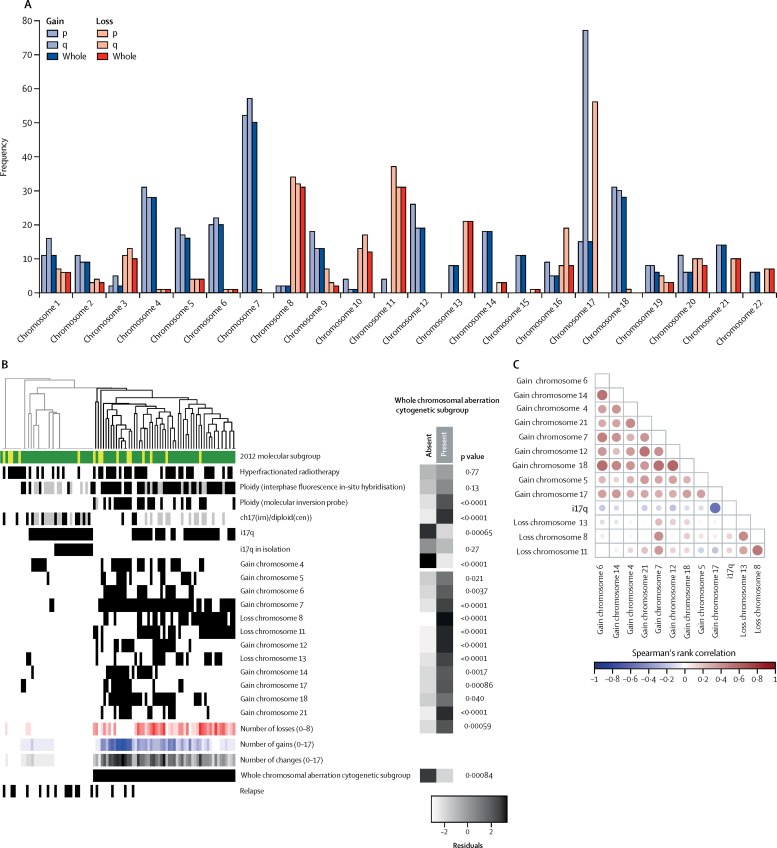
Identification of two cytogenetically distinct subgroups within non-WNT/non-SHH standard-risk medulloblastoma All 91 patient samples with non-WNT/non-SHH standard-risk medulloblastoma available from HIT-SIOP PNET 4 cohort are shown. (A) The frequency of p, q, and whole chromosome gains and losses for all autosomal chromosomes. (B) Unsupervised hierarchical clustering of chromosomal features. Grey indicates missing data. Residuals from χ^2^ indicate where whole chromosomal aberration cytogenetic group enrichment has occurred (darker shades of grey indicate stronger relationships), alongside p values from Fisher's exact tests. Total numbers of whole chromosomal losses (red), gains (blue), and changes (black) are shown. Increasing colour intensity indicates a larger number of changes. Chromosomal changes with incidence >15% are shown. We defined whole chromosomal aberration cytogenetic groups by hierarchical clustering. Green represents Group4 medulloblastoma and yellow represents Group3 medulloblastoma. (C) Correlation plot for recurrent (>15% incidence) cytogenetic changes. Circle area is proportional to the strength of correlation, with positive correlations shown in red and negative correlations shown in blue.

The 91 (67%) non-WNT/non-SHH tumours in the HIT-SIOP PNET 4 cohort of 136 were characterised by a higher incidence of whole chromosomal aberrations (eg, chromosome 7 gain, and chromosome 8 and 11 loss; mean 5·3 [SD 4·81] whole chromosomal aberrations per case for non-WNT/non-SHH *vs* 1·82 [SD 2·56] for WNT and 1·76 [SD 2·00] for SHH; Figure 1, Figure 3, appendix p 9), but isolated chromosome arm alterations were rare, with the exception of i17q (56 [62%] of 91 non-WNT/non-SHH medulloblastomas). However, 16 (18%) of 91 cases had no whole chromosomal aberrations (Figure 1, Figure 2). As expected, we observed structural cytogenetic (eg, i17q) and focal aberrations (including *MYCN* amplifications, *OTX2, CCND2*, and 18q12 [*TPTE2*] gains or amplifications, *SNCAIP* duplications, and 13q11–12 [*SETBP1*] loss; figure 1; appendix pp 11–12). Moreover, previously reported prognostic factors (*MYCN* amplification, i17q alterations, and subtotal resection)^11^, ^18^ were not associated with worse event-free survival (figure 2; appendix p 15), while the observed cohort-wide prognostic significance of whole chromosomal aberrations was maintained in this subgroup (figure 2).

We next investigated whether the observed molecular heterogeneity within the 91 non-WNT/non-SHH medulloblastoma tumours could inform its biological basis and clinical behaviour. Through unsupervised hierarchical cluster analysis of recurrent whole chromosomal aberrations, we identified two clinically and biologically distinct subgroups of tumours (figure 3). The first cytogenetic group was strongly associated with a pattern of i17q in isolation, diploid karyotypes, few recurrent whole chromosomal aberrations, and more relapses (p=0·00084). The second cytogenetic group was characterised by a spectrum of multiple recurrent and co-incident whole chromosomal aberrations (figure 3) and aneuploidy (p<0·0001; appendix p 13), and was associated with fewer relapses.

**Figure 4:**
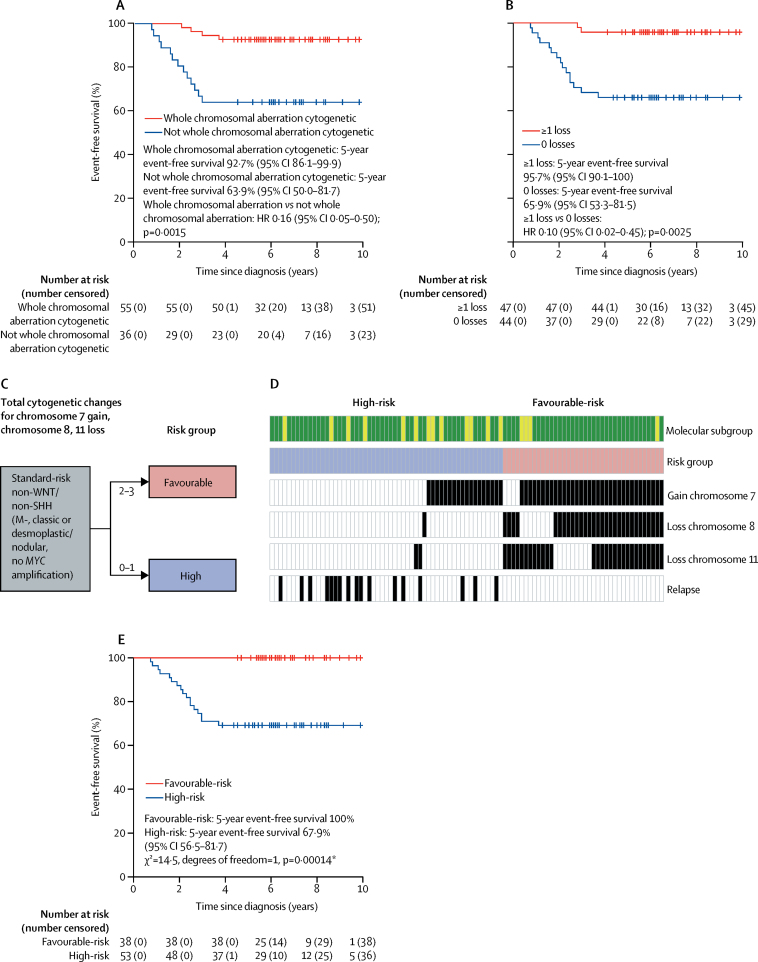
Whole chromosomal aberration-derived risk stratification schemes for non-WNT/non-SHH medulloblastomas All 91 available samples from patients in the HIT-SIOP PNET 4 cohort with non-WNT/non-SHH standard-risk medulloblastoma are shown. Event-free survival per (A) whole chromosomal aberration cytogenetic subgroup and (B) recurrent whole chromosomal losses (0 *vs* 1 or more changes). (C) Proposed optimally performing risk stratification model, with the two identified risk groups. (D) Incidence and distribution of prognostically relevant chromosomal changes. For molecular subgroup, green indicates Group4 and yellow indicates Group3. For risk group, blue indicates high-risk and red indicates low-risk. Black and white indicate presence or absence of a feature, respectively. (E) Event-free survival by the scheme shown in part C. HR=hazard ratio. *HR estimates for favourable-risk *vs* high-risk were not possible due to the group with no events. p value reported from log-rank test.

Whole chromosomal aberrations within non-WNT/non-SHH medulloblastoma samples were associated with improved 5-year event-free survival (figure 4). 55 (60%) of 91 non-WNT/non-SHH tumours had multiple recurrent and co-incident whole chromosomal aberrations and showed favourable outcomes compared with those without whole chromosomal aberrations (HR 0·16, 95% CI 0·05–0·50; p=0·0015; figure 4). The total number of whole chromosomal aberrations in a given tumour was prognostic for event-free survival. When different whole chromosomal aberration numbers were assessed, time-dependent AUC analysis identified 0 versus 1 or more recurrent whole chromosomal losses as the best discriminator of outcome (figure 4; appendix p 14). However, event-free survival was not only dependent on the total numbers of whole chromosomal aberrations. Analysis of the prognostic effect of specific whole chromosomal aberrations in individual chromosomes showed that chromosome 7 gain (HR 0·15, 95% CI 0·04–0·51, p=0·0025), chromosome 8 loss (HR calculation not possible because of group with no events; p=0·0014 for log-rank test), and chromosome 11 loss (HR 0·10, 95% CI 0·01–0·79, p=0·029) represented the most significant specific whole chromosomal aberrations (appendix pp 15–16).

Through assessment of event-free survival models for non-WNT/non-SHH medulloblastoma samples within the HIT-SIOP PNET 4 cohort, we identified at least two of the following—chromosome 7 gain, chromosome 8 loss, and chromosome 11 loss—as the optimally performing risk stratification scheme (appendix pp 14–15), outperforming other cytogenetic schemes and trial-based models, such as the SIOP PNET 5 MB trial model, in this patient group (figure 4; appendix p 14). This model, based on combinations of chromosome 7 gain, chromosome 8 loss, and chromosome 11 loss, stratified 38 (42%) of 91 non-WNT/non-SHH medulloblastomas as being favourable risk, with a 5-year event-free survival of 100%, (*vs* 68%, 95% CI 56·5–81·7 for high-risk tumours; p=0·00014 for log-rank test; figure 4). Further analysis of the high-risk patient group (53 [58%] of 91 patients), showed that 5-year event-free survival was equivalent between patients treated with hyperfractionated therapy or standard radiotherapy (HR 0·52, 95% CI 0·2–1·4, p=0·20 for Wald test; p=0·19 for log-rank test), consistent with findings from the overall HIT-SIOP PNET 4 trial cohort (data not shown).^14^

**Figure 5:**
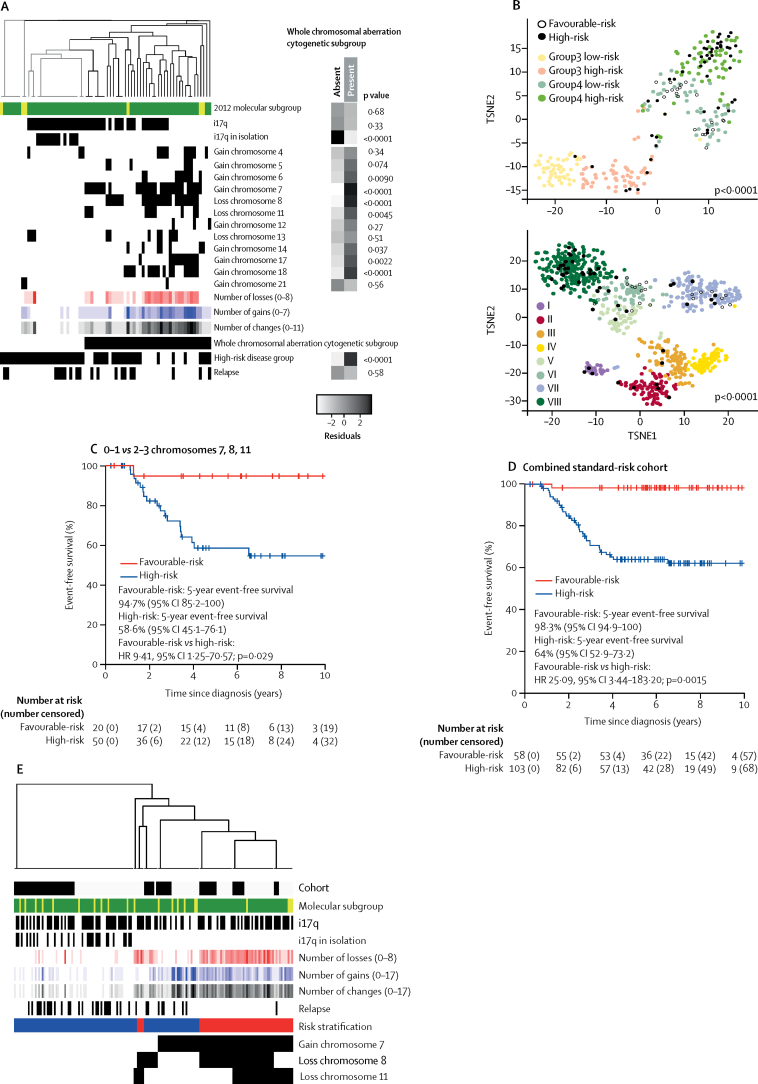
Validation of the whole chromosomal aberration-derived subgroups and risk stratification schemes All samples from the independent cohort of non-WNT/non-SHH-medulloblastoma (n=70) are shown in A and B. (A) Unsupervised clustering of chromosomal features by relevant chromosomal aberration cytogenetic subgroups. Residuals from χ^2^ tests indicate where whole chromosomal cytogenetic group enrichment has occurred. Darker shades of grey indicate stronger relationships. p values are from Fisher's exact tests. Total numbers of whole chromosomal losses (red), gains (blue), and changes (black) are shown. Increasing colour intensity indicates a higher number of changes. (B) Relationship of whole chromosomal aberration-defined risk groups to novel Group3 and Group4 disease subtypes. The standard-risk medulloblastoma validation cohort is indicated by filled and open circles according to risk, with relationship to the Schwalbe and colleagues^6^ and Northcott and colleagues^12^ cohorts shown by *t*-distributed stochastic neighbour embedding plots. (C) Event-free survival by whole chromosomal aberration-defined risk scheme. (D) Pooled analysis of event-free survival in the molecularly characterised HIT-SIOP PNET 4 cohort and validation cohort, stratified by derived whole chromosomal aberration-defined risk scheme. (E) Patterns of prognostically important cytogenetic changes in the combined cohort. The validation cohort is labelled black. Risk stratification is labelled red (favourable-risk) and blue (high-risk). HR=hazard ratio. TSNE=*t*-distributed stochastic neighbour embedding.

We tested the reproducibility of our findings in an independent cohort of 70 non-WNT/non-SHH medulloblastomas, which matched the clinical and demographic characteristics of our HIT-SIOP PNET 4 standard-risk medulloblastoma cohort, collected from 1990 to 2014 (table, figure 5). The median event-free survival for these patients was 5·6 years (IQR 3·1–8·1).

The characteristics, incidence, and associated event-free survival outcomes of the identified whole chromosomal aberration-defined subgroups were recapitulated (figure 5; appendix p 17). Our proposed whole chromosomal aberration signature-based model represented the best performing risk stratification scheme (figure 5; appendix p 18). The favourable-risk whole chromosomal aberration signature, defined by chromosome 7 gain, chromosome 8 loss, and chromosome 11 loss, was observed within multiple novel methylation subgroups, and was significantly associated with MB_Group4-LowRisk_^6^ and Group3 and Group4 subtypes VI and VII^12^ (p<0·0001, appendix p 18). By contrast, the high-risk group was significantly associated with MB_Group4-HighRisk_^6^ and subtype VIII^12^ (p<0·0001, figure 5; appendix p 18). When we considered our event-free survival models in Group4 patients alone, we found equivalent prognostic relationships in both the HIT-SIOP PNET 4 and validation cohorts (appendix pp 14, 19–20).

A pooled analysis applied the validated whole chromosomal aberration signature-based risk-stratification model to the merged non-WNT/non-SHH medulloblastomas from the HIT-SIOP PNET 4, and validation cohorts (n=161) and classified 58 (36%) non-WNT/non-SHH medulloblastomas as favourable-risk and 103 (64%) as high-risk; 5-year event-free survival was 98·3% (95% CI 94·9–100) in the favourable-risk group *vs* 64% (52·9–73·2) in the high-risk group (HR 25·09, 95% CI 3·44–183·20; p=0·0015; figure 5). Together with established favourable-risk WNT medulloblastomas in patients younger than 16 years (20 [15%] of 136 patients; appendix pp 5–6) and *TP53*^wild-type^ SHH medulloblastomas (11 [8%] of 136 tumours; appendix pp 7–8), these newly identified chromosomal signatures identified 69 (51%) of 134 (two SHH tumours had unknown *TP53* status and were therefore excluded from the calculation) molecularly characterised patients with medulloblastoma from the HIT-SIOP PNET 4 cohort with a favourable prognosis (5-year event-free survival of 100%).

## Discussion

Implementation of enabling technologies (MS-MIMIC and molecular inversion probe assay) allowed us to systematically assess the molecular pathology of the standard-risk medulloblastoma clinical group within the HIT-SIOP PNET 4 patient cohort. To our knowledge, no equivalent multicentre, prospective investigations of standard-risk medulloblastoma have been reported. Although wider, retrospective medulloblastoma datasets are available, these typically lack the full clinical and molecular annotation necessary to define the standard-risk medulloblastoma group. The standard-risk medulloblastoma group displayed distinct demographics versus disease-wide cohorts.^8^, ^9^, ^10^, ^11^ WNT and Group4 subgroups were enriched within the standard-risk medulloblastoma cohorts because of the absence of clinicomolecular high-risk features in standard-risk disease.

The favourable prognosis of patients with WNT medulloblastoma was confirmed in patients from the HIT-SIOP PNET 4 cohort who were younger than 16 years at diagnosis. However, patients older than 16 years did not share this good prognosis, consistent with previous reports.^15^, ^23^ Together, these data do not support therapy de-escalation in patients with WNT medulloblastoma older than 16 years of age. Patients with SHH medulloblastoma without *TP53* mutations (SHH-*TP53*^wild-type^) or chromosome 17p loss similarly had a favourable prognosis. These data validate independent previous findings^6^, ^7^ and support the eligibility of these patients for de-escalated or targeted therapies (eg, *SMO* inhibitors).^24^

Development of biomarker-driven treatment strategies for the large remaining group of patients with non-WNT/non-SHH disease represents the largest ongoing challenge for standard-risk medulloblastoma. In the absence of high-risk features,^5^ these patients had a 5-year event-free survival of 81% (95% CI 74–90) in the HIT-SIOP PNET 4 trial. As described in this Article, non-WNT/non-SHH medulloblastoma tumours have few recurrent mutations, and structural chromosomal abnormalities are the most common genomic features.^8^, ^9^, ^10^ When comparing Group4 and Group3 tumours, we found around 90% overlap of chromosomal alterations between the two subgroups and equivalent event-free survival. Coupled with evidence supporting their shared underlying biological mechanisms,^6^, ^12^ we considered Group3 and Group4 tumours together in our analysis. We identified two biologically and clinically distinct non-WNT/non-SHH medulloblastoma groups. The first group was a cytogenetically quiet, high-risk group associated with diploid genomes, many with i17q as the sole defining genomic feature. These tumours provide a wider biological context for the poor-risk group of patients with non-WNT disease with chromosome 17p or q defects in a diploid background (chr17(im)/diploid(cen)), previously identified by interphase FISH in this cohort.^15^ The second group was large and defined by multiple, co-occurring whole chromosomal aberrations, common polyploidy, and improved relative outcomes.

In this whole chromosomal aberration group, using multivariable event-free survival analysis and risk modelling, we deduced a whole chromosomal aberration signature (two or more of chromosome 7 gain, chromosome 8 loss, and chromosome 11 loss), which best defined patients with non-WNT/non-SHH medulloblastoma with favourable prognosis. We validated these findings in an independent demographically matched standard-risk medulloblastoma cohort, and they were reproducible when Group4 patients were considered in isolation. This whole chromosomal aberration signature was detected within a number of novel methylation subgroups within non-WNT/non-SHH medulloblastoma, and associated with the low-risk MB_Group4-LowRisk_,^6^ and Group3 and Group4 subtypes VI and VII.^12^ By contrast, the high-risk isolated i17q diploid group was associated with high-risk MB_Group4-HighRisk_^6^ and subtype VIII.^12^ These associations suggest common biological phenotypes and evaluation of their relative contributions to risk stratification could be investigated in future clinically controlled studies.

Biologically and clinically significant whole chromosomal phenotypes are a notable feature of childhood malignancies other than medulloblastoma. Characteristic patterns of non-random whole chromosomal aberrations in neuroblastoma (so-called whole-chromosomal changes phenotype; more than two whole chromosomal aberrations)^25^, ^26^ and high hyperdiploid acute lymphoblastic leukaemia (so-called high-hyperdiploidy phenotype [HeH]; 51–65 chromosomes)^27^ define tumour subgroups with favourable prognoses. Additionally, choroid plexus papillomas and adult infratentorial ependymomas (posterior fossa ependymoma type B) are characterised by multiple whole chromosomal abberations.^1^ Overall, whole chromosomal aberration signatures are associated with a low number of single nucleotide mutations.

This common involvement of whole chromosomal aberration signatures provides strong impetus to understand the underlying molecular pathomechanisms, including errors in mitotic control, chromosome segregation, and function of the spindle apparatus. Although beyond the scope of this study, investigation of associated biology (eg, gene-expression profiles, pathway involvements, and driver events) and the involvement of specific chromosomes (ie, chromosomes 7, 8, and 11), is essential to improve understanding and therapeutic targeting. Potential opportunities include agents that target the spindle apparatus or mitotic control. For instance, vincristine (a component of medulloblastoma treatment regimens) directly targets the spindle apparatus, and the excellent whole chromosomal aberration signature-associated outcomes might be explained by high sensitivity to such treatments. Indeed, the association between HeH acute leukaemia and chemosensitivity associated with increased DNA content has been long established.^28^

This study has some limitations. The developed risk stratification scheme applies only to non-infant, standard-risk medulloblastoma treated with standard multimodal therapies. Children younger than 4 years, patients treated with chemotherapy only, and high-risk patients require independent assessment and development of appropriate risk stratification schemes. However, our biomarker-driven risk stratification schemes for standard-risk medulloblastoma are readily testable in routine molecular diagnostic practice and, following their validation in independent clinically controlled and biomarker-defined cohorts, could form the basis of international clinical trials aimed at improving outcomes.

In summary, our molecular pathological characterisation of the HIT-SIOP PNET 4 cohort identified and independently validated a whole chromosomal aberration signature-defined subgroup of non-WNT/non-SHH medulloblastomas associated with good prognosis. Combination of these newly defined subtypes with the favourable-risk WNT and SHH medulloblastomas validated in our study redistributed around 50% of patients with standard-risk medulloblastoma into a favourable-risk group, who could benefit from reduced-intensity therapies aimed at maintaining overall survival while reducing treatment-associated toxicities and late effects. Patients not classified into this favourable-risk group had a 5-year event-free survival of around 60% and should be considered high risk. In the HIT-SIOP PNET 4 cohort, this model compared favourably with published and currently accepted risk stratification schemes (eg, Shih and colleagues^11^ and SIOP PNET 5 MB;^5^ appendix p 14) and redefines our understanding of biomarkers and disease risk within the previously clinically defined standard-risk medulloblastoma patient group.
