# Supplementary material for: Prognostic effect of whole chromosomal aberration signatures in standard-risk, non-WNT/non-SHH medulloblastoma: a retrospective, molecular analysis of the HIT-SIOP PNET 4 trial
Source: Lancet Oncol. 2018 Dec;19(12):1602–16. doi: 10.1016/S1470-2045(18)30532-1 (PMC6262170; doi:10.1016/S1470-2045(18)30532-1)
Supplement: Supplementary appendix [file mmc1.pdf]

THE LANCET
Oncology

Supplementary appendix

This appendix formed part of the original submission and has been peer reviewed. We post it as supplied by the authors.

Supplement to: GoschzikT, Schwalbe EC, Hicks D, et al. Prognostic effect of whole chromosomal aberration signatures in standard-risk, non-WNT/non-SHH medulloblastoma: a retrospective, molecular analysis of the HIT-SIOP PNET 4 trial. *Lancet Oncol* 2018; published online Nov 1. http://dx.doi.org/10.1016/S1470-2045(18)30532-1.

Prognostic effect of whole chromosomal aberration signatures in standard-risk, non-WNT/non-SHH medulloblastoma: a retrospective, molecular analysis of the HIT-SIOP PNET 4 clinical trial

T Goschzik, E C Schwalbe, D Hicks et al.

Appendix

Supplementary methods

Study design and participants

In this retrospective analysis, we assessed a subset of samples from the HIT-SIOP PNET 4 clinical trial (n=136; 'clinical and molecular cohort'). The trial investigated treatment outcomes using either hyper-fractionated radiotherapy (HFRT) or standard delivery (STRT).[1] Patients with LCA histology were excluded during the clinical trial and from this analysis. Additionally, due to their reported poor prognosis, tumours with a *MYC* amplification were removed. Patients with STR tumours[2] and/or *MYCN* amplification were retained to assess their prognostic significance in a clinically-controlled cohort.[3–5]

Diagnostic samples were collected from 2001–2006 and underwent full central clinical review. Patients were aged from 3–20 years at diagnosis (median 9 years). For this study, updated survival information was available compared to this cohort's initial descriptions,[1,5] with median follow-up of 6·6 years (IQR 5·6-8·5 years). The demographic features of the clinical and molecular cohort were comparable with the whole trial cohort, and prognostic features were consistent. Importantly, in accordance with the whole trial cohort, there was no survival difference between HFRT and STRT (figure 2A). We validated our findings in a demographically matched, independent, retrospective cohort of SR non-WNT/non-SHH-MB (n=70). This matched cohort was selected from primary MB collected from UK Children's Cancer and Leukaemia Group (CCLG) and SIOP-Europe associated treatment centres, aged from 4–20 years at diagnosis, with metastatic stage M0, non-amplified MYC and in receipt of cranio-spinal irradiation. 9 samples were recruited from 1990–2000, 41 from 2000–2010 and 20 from 2010–present. 53 samples were gross-totally resected, 20 sub-totally resected and two had unknown resection status. Chang's criteria were used to assign metastatic stage.6 Tumours were classed as STR if their residuum following excision exceeded 1.5cm^2. *MYC* and *MYCN* amplification were assessed as previously described.[3,5] All samples were collected with written, informed consent as part of the CCLG-approved biological study BS-2007-04. Tumour investigations were done with approval from Newcastle/North Tyneside Research Ethics Committee, study reference 07/Q0905/71.

Molecular subgrouping using MS-MIMIC

Since the HIT-SIOP PNET 4 trial protocol predated the identification of molecular subgroups of medulloblastoma, it did not mandate the collection of materials for molecular subgrouping. However, minute remnant materials (nuclear cytospin preparations), originally intended for FISH and unsuitable for assessment of subgroup using conventional approaches (DNA methylation array[7] and/or Nanostring[8]), were available.

Using our recently described MS-MIMIC assay,[9] which assigns molecular subgroup of medulloblastoma using minimal DNA methylation signatures in limited materials, we were able to confidently assign subgroup for 136 tumour samples for which remnant FFPE materials were available with matched molecular inversion probe-derived copy number profiles. The average DNA yield for use was ~50ng, for full details regarding quality and quantity of DNA derived from FFPE tissue sections and cytospin nuclear preparations (see Schwalbe et al.[9]).

Molecular inversion probe (MIP) array

To identify copy number gains and losses, we used a MIP array including 335,000 inversion probes (Version v2·0, Affymetrix, Santa Clara, USA) with a median probe spacing of 2·4 kb. The MIP array also contains probes for 541 frequent somatic cancer mutations and was performed as previously described.[10] In brief, all 335,000 probes contain two genomic homology regions each flanking a SNP site. After annealing to the DNA, the gaps are filled and ligated. Exonucleases digest remaining non-circularized probes. After cleavage the now inverted probes are amplified by PCR using universal primers and labelled with fluorescent molecules and hybridised to oligonucleotide chip arrays. Raw MIP data was analysed using the Nexus Copy Number 7·0 Discovery Edition software (BioDiscovery, El Segundo, USA). BioDiscovery's SNP-FASST2-Segmentation algorithm was used to make copy number and loss of heterozygosity (LOH) calls. When at least 90% of the probe signals from both chromosome arms were above/below the defined threshold, whole chromosomes were counted as gained/lost. For acrocentric chromosomes (13, 14, 15, 21, and 22) only q-arms were analysed and were counted as whole chromosomal gains/losses.

Ploidy was determined by using the allele ratio data from the MIP assay. GISTIC (Genomic Identification of Significant Targets in Cancer) analysis was used to identify significant focal chromosomal aberrations from random background (p level: 0·05).[11] GISTIC analyses uncovered significantly altered chromosomal regions in each genetically defined MB entity and variant (see appendix pp 10–12).

Statistical and survival analysis

Event free survival (EFS) was defined as the time from receipt of surgery to first event (progression or relapse), or date of last follow-up. Patients whose follow-up time exceeded 10 years were right-censored at 10 years. We constructed Kaplan-Meier curves and compared patient groups with log-rank tests.

Using hierarchical clustering, we clustered non-WNT/non-SHH-MB by their recurrent (*i.e.* incidence >15%) autosomal WCAs (having excluded WCAs on the sex chromosomes), using a binary distance measure and average linkage. The co-incidence of recurrent WCAs was visualised using the R package *corrplot*. After molecular subgrouping, we observed comparable cytogenetic changes (appendix p 9) and outcomes (figure 2C) between non-WNT/non-SHH-MB tumours. Consequently, given this and the emerging evidence of their shared biology,[3,12] we considered these groups together in subsequent survival analyses.

Using Cox modelling, we tested the prognostic power of clinical markers (female sex *vs* male sex, HFRT *vs* STRT, STR *vs* fully-resected disease, *MYCN* amplification *vs* no amplification, and desmoplastic / nodular (DN) *vs* classic (CLA) histology) and presence/absence of recurrent WCA. Finally, also using Cox modelling, we tested patient stratifications defined by varying cutoffs for total WCAs, WCA gains and WCA losses. Additionally, we derived pragmatic assignments of patient risk by combining WCAs significant in univariate testing. We assessed the predictive power of the identified groups by calculating total area under the curve (AUC), sensitivity and specificity at 5 years, using time-dependent Receiver Operating Characteristic (ROC) curves with the R package *timeROC*. We verified the proportionality assumption for Cox modelling using scaled Schoenfeld residuals.

The validation cohort was run on the Illumina 450k DNA methylation microarray and chromosome arm gain/loss and *MYC/MYCN* amplification assessed as previously described.[3] Since copy-number was estimated using different technologies between the primary (MIP array) and validation cohorts (DNA methylation array), we tuned copy-number calling to maximise inter-platform agreement on a panel of tumours that had matched MIP and 450k data. Subsequently, using the validation cohort, we performed equivalent clustering of recurrent WCAs and tested the stratification schemes developed with the HIT-SIOP PNET 4 SR non-WNT/non-SHH-MB cohort. Finally, in order to better understand the nature of the identified risk groups, we classified the validation cohort according to the recently published refinements of substructure within non-WNT/non-SHH-MB.[3,12]

Briefly, we used previously developed methylation-dependent classifiers,[3] or recapitulated the underpinning analysis (tSNE clustering / DBSCAN) on a combined cohort that included tumours from the original study in addition to the validation cohort.[12]

The significance threshold was set at p<0·05 for all statistical tests in this study, unless otherwise stated. Significance of association was assessed using Fisher's exact tests; the strength of associations were visualised using chi-squared test residuals. Statistical or bioinformatic analyses were implemented using R (version 3·4·2).

Supplementary table 1: Participating countries of the pan-European HIT-SIOP PNET 4 clinical trial with number of patients (total n=136)

Country	Lead Clinicians	n	%
Germany	R. Kortmann,<br>S. Rutkowski	85	62·5
Italy	M. Massimino<br>L. Gandola	16	11·8
France	F. Doz<br>C. Carrie	13	9·6
Sweden , Norway	B. Lannering	9	6·6
UK	B. Pizer<br>R. Taylor	6	4·4
Spain	A. Navajas	6	4·4
Netherlands	R. Reddingius<br>F. Oldenburger	1	0·7

Supplementary figure 1: Molecular subgroup and cytogenetic analysis of the HIT-SIOP PNET 4 clinical trial

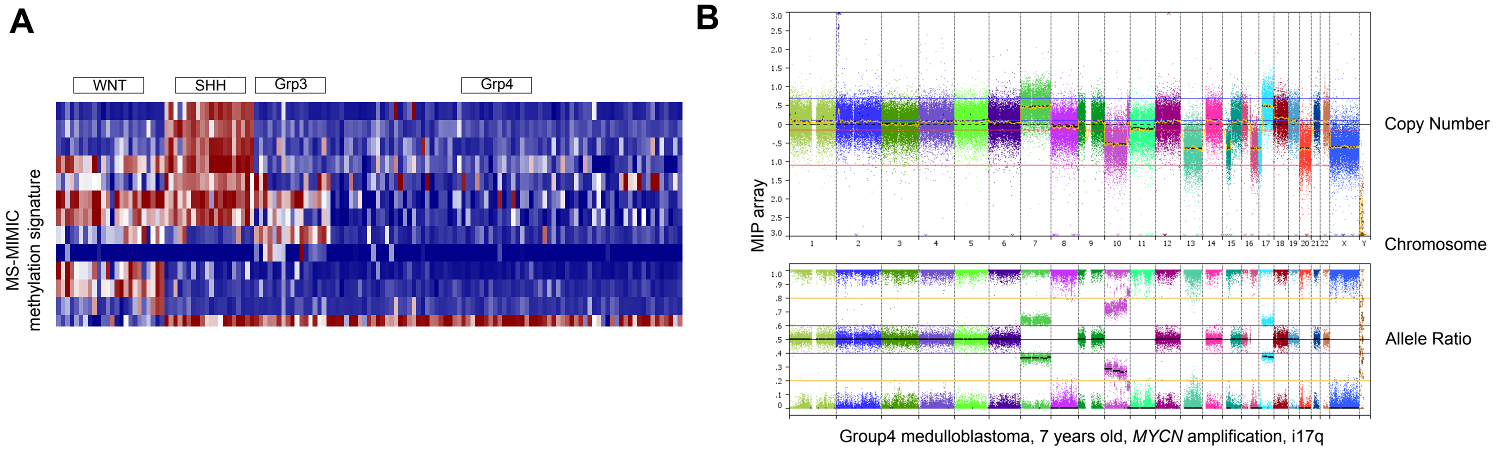

(A) MS-MIMIC DNA methylation signature demonstrates subgroup-specific DNA methylation profiles at signature loci. Heatmap shows subgroup-informative loci, with red indicating hypermethylation and blue, hypomethylation. (B) MIP array enables identification of both broad and focal copy number changes as well as assessment of loss of heterozygosity. An example Group4, *MYCN* amplified tumour is shown.

Supplementary figure 2: WNT-MB subgroup analysis in the HIT-SIOP PNET 4 clinical trial

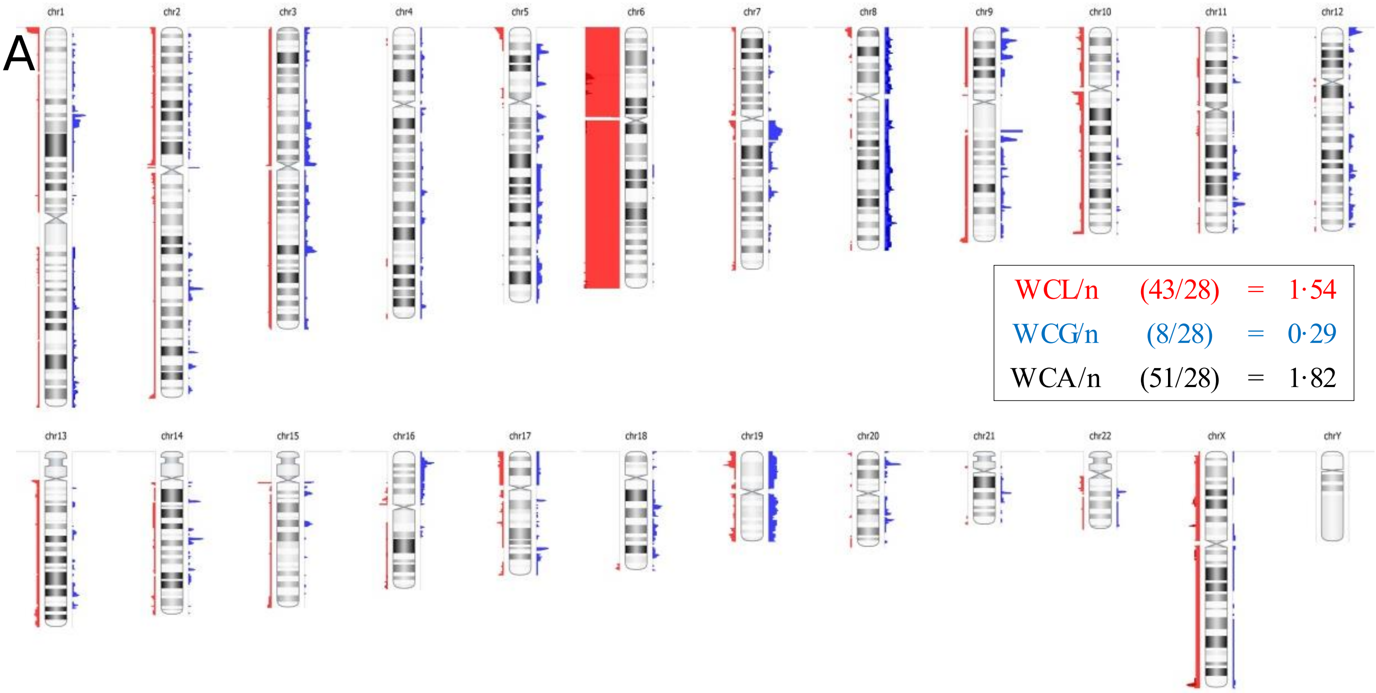

B

Variable	Categories	n; events SR PNET4 WNT	p value SR PNET4 WNT
Age	4–15 years<br>16–20 years	20; 0<br>8; 3	**0·00050**
Gender	Male<br>Female	10; 2<br>18; 1	0·20
Residual tumour	GTR<br>STR	25; 2<br>3; 1	0·26
Treatment	STRT<br>HFRT	20; 2<br>8; 1	0·66
CTNNB1 mutation	Yes<br>No	26; 2<br>2; 1	0·11
Monosomy 6	Yes<br>No	25; 2<br>3; 1	0·15

C

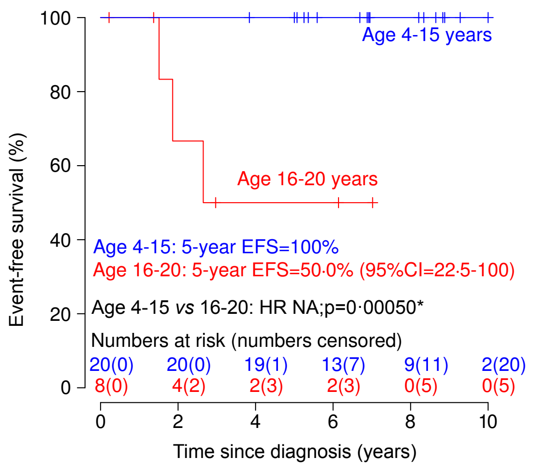

*Estimates of HR for 4-15 year-old group not possible due to no events. P value from log rank is shown.

Supplementary figure 2 (cont.)

D

Relapsed cases

	Gender	Age (years)	Event	Residual tumour	Treatment	*CTNNB1* mutation	Monosomy 6	Histology
1	Female	16	Yes	STR	STRT	No	Yes	CLA
2	Male	16	Yes	GTR	STRT	S33F	No	CLA
3	Male	16	Yes	GTR	HFRT	S33Y	Yes	CLA

E

Excluded cases

	Gender	Age (years)	Event	Residual tumour	Treatment	*CTNNB1* mutation	Monosomy 6	Exclusion criteria
1	Female	9	Yes	GTR	STRT	ΔLD	NA	no *MYC* data
2	Male	6	No	GTR	STRT	G34R	Yes	LCA
3	Male	6	No	GTR	STRT	S33C	Yes	LCA
4	Female	9	No	GTR	HFRT	G34V	Yes	LCA

F

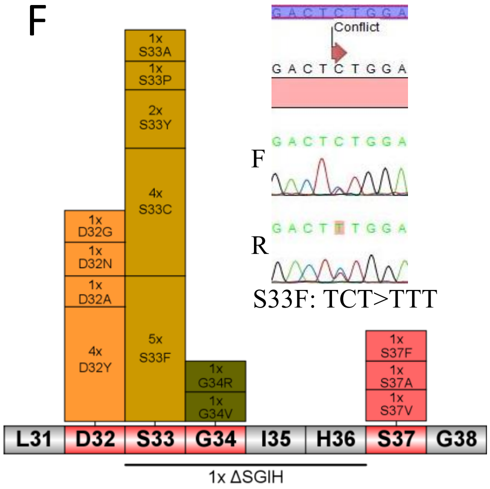

(A) Summary plot of WNT copy number aberrations (n=28 tumours) from molecular inversion probe (MIP) array. Blue bars, gains; red bars, losses. Thickness of bars indicates frequency of alterations. (B) Distribution and prognostic significance of clinical and molecular variables (n=28 patients); p values, log-rank test. (C) Kaplan-Meier EFS plot for age at diagnosis (<16 years vs ≥16 years). (D) Demographics, clinical, and molecular data from all relapsed WNT patients. (E) Demographics, clinical, and molecular data from WNT cases excluded from the study cohort due to missing MYC status information (1) or large-cell/anaplastic (LCA) histology (2–4). (F) *CTNNB1* mutations and deletions in WNT tumours. A representative Sanger sequencing electropherogram (S33F mutation; forward (F) and reverse (R)) is shown. Abbreviations: WCL, whole chromosomal losses; WCG, whole chromosomal gains; WCA, whole chromosomal aberrations; GTR, gross-total resection; STR, sub-total resection; STRT, standard fractionated radiotherapy; HFRT, hyperfractionated radiotherapy; CLA, classic; NA, not analysed; Δ, deletion.

Supplementary figure 3: SHH-MB subgroup analysis in the HIT-SIOP PNET 4 clinical trial

A

Variable	Categories	n; events SR PNET4 SHH	p value SR PNET4 SHH
Gender	Male<br>Female	10; 2<br>7; 2	0·70
Histology	DN<br>CLA	14; 4<br>3; 0	0·31
Residual tumour	GTR<br>STR	15; 4<br>0; 0	-
Treatment	STRT<br>HFRT	4; 0<br>13;4	0·29
17p13·1 loss (*TP53* loss)	Yes<br>No	6; 4<br>11; 0	**0·0022**
TP53 mutation	Yes<br>No	4; 3<br>11; 0	**0·0011**
Chr14 loss	Yes<br>No	2; 2<br>15; 2	**0·0074**
MYCN ampl.	Yes<br>No	0; 0<br>17;4	-
GLI2 ampl. or chr14 loss (Shih SHH model[4])	Yes<br>No	3; 3<br>14; 1	**0·00067**

B

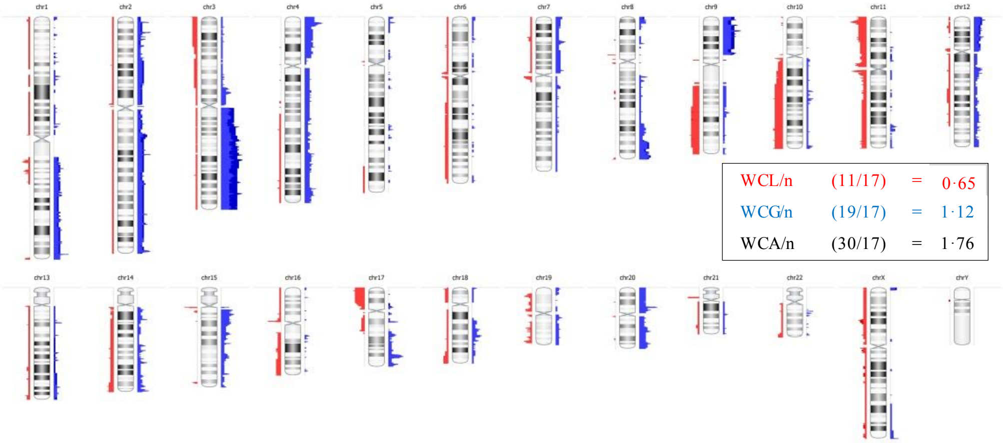

C

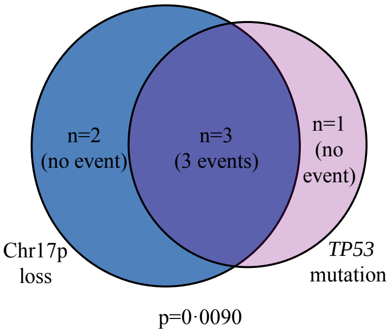

Supplementary figure 3 (cont.)

D

	Gender	Age (years)	Histology	Treatment	Residual tumour	Event	MIP data	17p13.1 loss (*TP53* loss)	*TP53* mutation status	*MYCN* ampl.	Shih SHH model[4]	Comment
1	Male	14	CLA	HFRT	NA	No	Yes	No	NA	No	-	
2	Male	11	DN	HFRT	GTR	No	Yes	No	WT	No	-	
3	Male	16	DN	HFRT	GTR	No	Yes	No	WT [R213R (CGA>CGG; exon 6)]	No	-	
4	Female	7	DN	HFRT	GTR	No	Yes	No	WT	No	-	
5	Male	19	DN	HFRT	GTR	No	Yes	No	WT	No	-	
6	Female	15	DN	STRT	GTR	No	Yes	No	WT	No	-	
7	Male	15	DN	HFRT	GTR	No	Yes	No	WT	No	-	
8	Female	8	CLA	HFRT	GTR	No	Yes	No	WT	No	-	
9	Female	15	DN	STRT	GTR	No	Yes	No	WT	No	-	
10	Male	3	DN	HFRT	NA	No	Yes	No	WT	No	-	
11	Male	12	CLA	HFRT	GTR	No	Yes	No	G154S (GGC>AGC; exon 5)	No	-	
12	Female	14	DN	STRT	GTR	No	Yes	Yes	WT	No	-	
13	Male	5	DN	STRT	GTR	No	Yes	Yes	WT	No	-	
14	Male	8	DN	HFRT	GTR	Yes	Yes	Yes	NA	No	Chr14 loss	
15	Male	7	DN	HFRT	GTR	Yes	Yes	Yes	V197M (GTG>ATG; exon 6)	No	-	
16	Female	11	DN	HFRT	GTR	Yes	Yes	Yes	R248W (CGG>TGG; exon 7)	No	*GLI2* ampl.	Li-Fraumeni patient
17	Female	13	DN	HFRT	GTR	Yes	Yes	Yes	R282G (CGG>GGG; exon 8)	No	Chr14 loss	
18	Female	11	DN	HFRT	GTR	No	No	No (iFISH)	NA	No	NA	no MIP
19	Female	9	CLA	HFRT	GTR	No	No	No (iFISH)	NA	No	NA	no MIP
20	Male	16	DN	HFRT	GTR	No	No	Yes (iFISH)	WT	No	NA	no MIP
21	Male	4	DN	STRT	GTR	No	No	NA	NA	NA	NA	Excluded
22	Male	14	LCA	STRT	GTR	No	Yes	No	WT	No	-	Excluded
23	Female	5	DN	HFRT	NA	Yes	No	NA	NA	NA	NA	Excluded
24	Female	6	LCA	HFRT	GTR	Yes	Yes	No	WT [P142P (CCT>CCC; exon 5)]	No	-	Excluded
25	Female	5	LCA	STRT	GTR	Yes	Yes	Yes	R273H (CGT>CAT; exon 8)	Yes	*GLI2* ampl.	Excluded

(A) Distribution and prognostic significance of clinical and molecular variables (n=17 patients); p values, log-rank test. (B) Summary plot of SHH copy-number aberrations (n=17 tumours) from molecular inversion probe (MIP) array. Blue bars, gains; red bars, losses. Thickness of bars indicates frequency of alterations. (C) Venn diagram showing cases with chr17p losses and/or *TP53* mutations. P value from chi-squared test. (D) Demographics, clinical, and molecular data from SHH cases. Patients with subgroup information only but without MIP data (18–20) are shown in light grey shading. Patients excluded from the study cohort due to LCA histology (22, 24, 25) or missing *MYC* data (21 and 23) are shown in dark grey shading. Abbreviations: DN, desmoplastic/nodular; CLA, classic; LCA, large-cell/ anaplastic; WCL, whole chromosomal losses; WCG, whole chromosomal gains; WCA, whole chromosomal aberrations; GTR, gross-total resection; STR, sub-total resection; STRT, standard fractionated radiotherapy; HFRT, hyperfractionated radiotherapy; NA, not analysed.

Supplementary figure 4: SR HIT-SIOP PNET 4 non-WNT/non-SHH-MB analyses

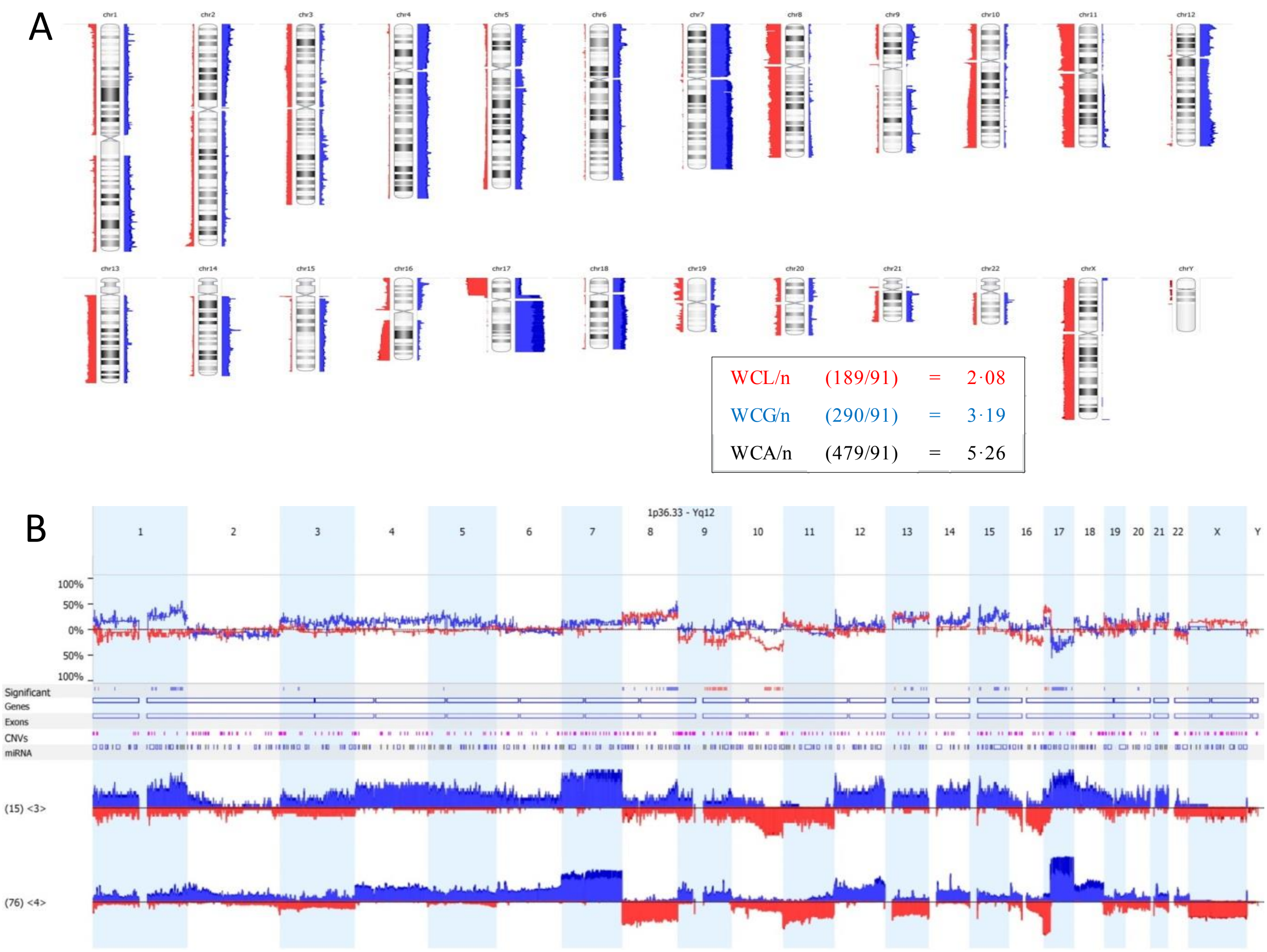

(A) Summary plot from SR HIT-SIOP PNET 4 non-WNT/non-SHH cases from molecular inversion probe (MIP) assay (n=91). Blue bars, gains; red bars, losses. Thickness of bars indicates frequency of alterations. (B) Comparison analysis using Nexus software from Group3 *vs* Group4 SR HIT-SIOP PNET 4 medulloblastoma samples. Group4 samples are set as baseline (p=0·005; differential threshold=25%). Abbreviations: WCL, whole chromosomal losses; WCG, whole chromosomal gains; WCA, whole chromosomal aberrations; CNVs, copy number variations.

Supplementary figure 5: SR HIT-SIOP PNET 4 GISTIC analyses

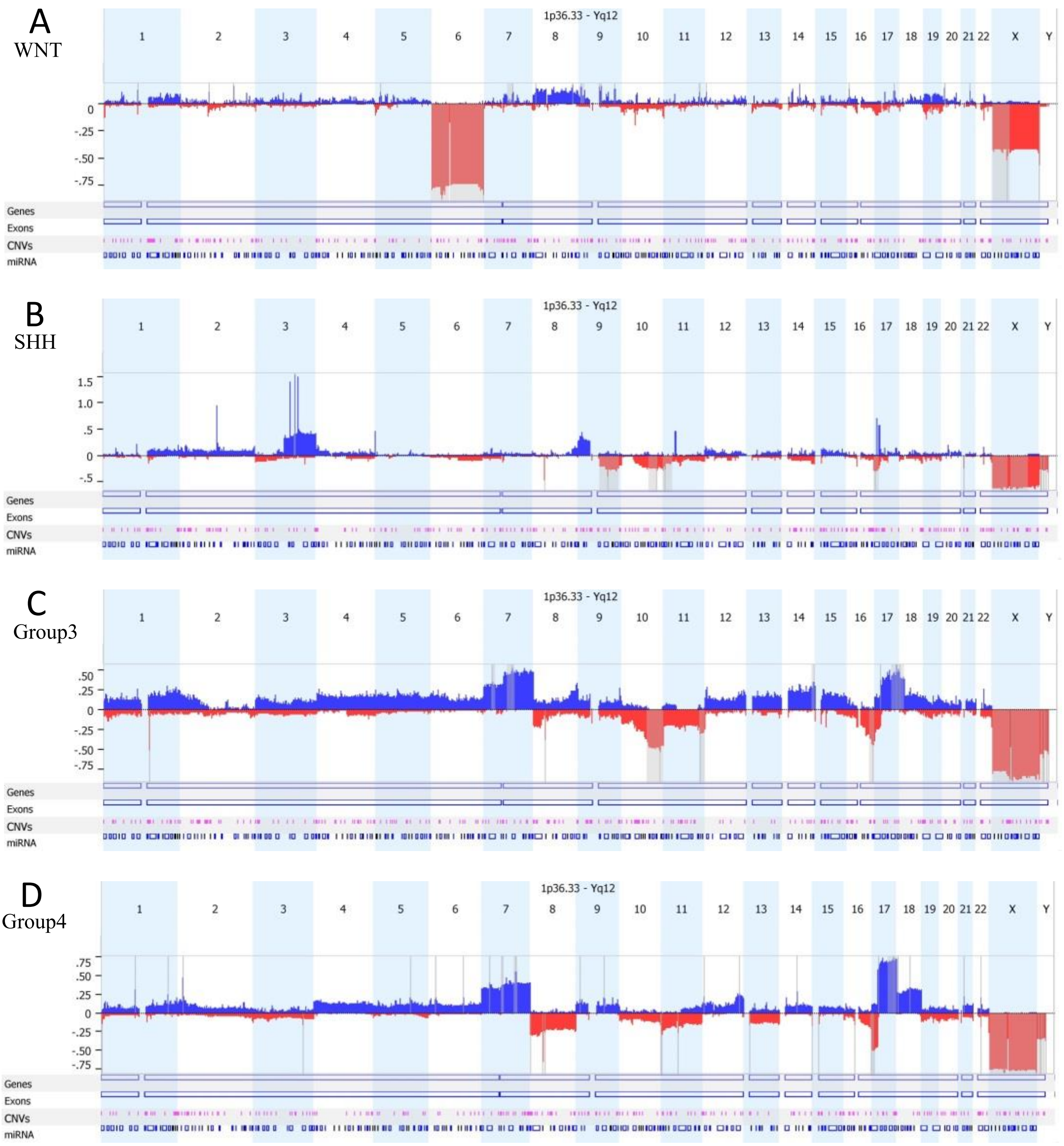

Supplementary figure 5 (cont.)

SR HIT-SIOP PNET 4 non-WNT/non-SHH-MB

E

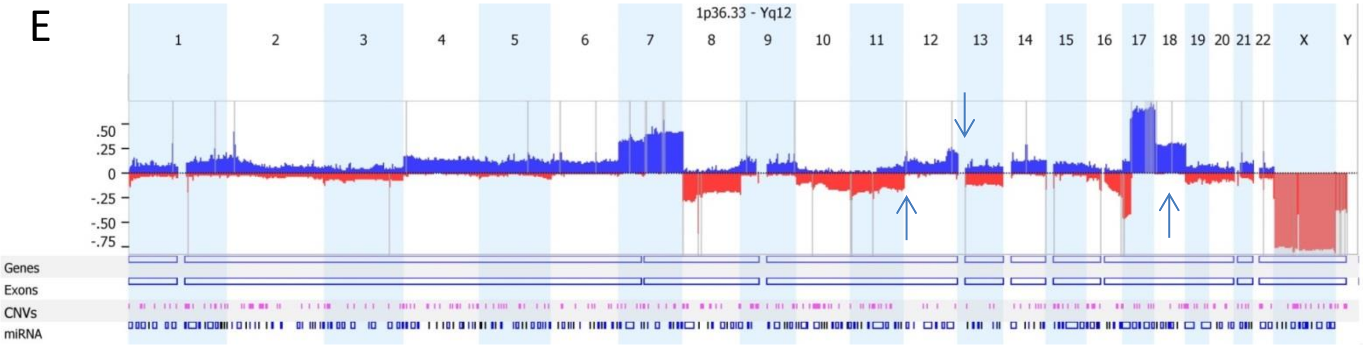

(A)–(E) GISTIC plots (Q-Bound cut-off: 0·05) of HIT-SIOP PNET 4 SR-MB samples. (A) WNT-MB (n=28). (B) SHH-MB (n=17). (C) Group3-MB (n=15). (D) Group4-MB (n=76). (E) Non-WNT/non-SHH-MB (Group3/4 combined) (n=91), detailed table of GISTIC peaks in appendix p 12. Arrows indicate GISTIC regions where only the focal regions but not the whole chromosomal aberrations reach significant values (log-rank test). In appendix p 12, these regions are labelled with red background colour. Significant GISTIC regions are shown in darker grey within extended GISTIC regions (light grey). Often GISTIC regions and extended regions are equal (only dark grey). Abbreviations: CNVs, copy number variations.

Supplementary table 2: Detailed GISTIC analysis of HIT-SIOP PNET 4 non-WNT/non-SHH-MB (from appendix p 11 (suppl. figure 5E))

Region	Extended Region	Type	Q-Bound	% of CNV Overlap	Chr. arm region (extended)	log-rank	n/events SR PNET4 non-WNT/non-SHH (91/17)	Key candidate gene(s)	No. of genes / CNV / miRNAs (in extended region)	Also found in Northcott et al. (Nature, 2012)[13]	Remarks	WCA log-rank (n;events)
chr1:110,227,064-110,243,644	chr1:110,227,064-110,243,644	CN Gain	p<0·0001	100	1p13·3	p=0·48	22/3	*GSTM1*	1 / 39 / 0			0·39 (gain) 11;1
chr1:216,753,408-216,906,408	chr1:216,692,358-216,911,490	CN Gain	p=0·0011	0	1q41	p=0·63	38/8	*ESRRG*	1 / 0 / 0	Yes (Grp3 + Grp4)		0·39 (gain) 11;1
chr2:16,076,116-16,090,840	chr2:15,853,565-16,180,515	CN Gain	p<0·0001	88·80	2p24·3	p=0·221	33/4	*MYCN*	3 / 4 / 0	Yes (Grp3 + Grp4)		0·79 (gain) 9;2
chr4:7,400,243-7,612,707	chr4:6,625,969-7,976,785	CN Gain	p=0·026	6·40	4p16·1	p=0·68	36/6	*S100P*	21 / 101 / 1		ns in Grp4 only	0·22 (gain) 28;3
chr5:121,703,802-121,789,638	chr5:121,644,804-121,907,549	CN Gain	p=0·0070	0	5q23·2	p=0·18	29/3	*SNCAIP*	2 / 1 / 0	Yes (Grp4)		0·51 (gain) 16;2
chr6:22,792,558-22,873,779	chr6:20,818,319-23,647,132	CN Gain	p=0·0038	0	6p22·3	p=0·16	41/5	*SOX4*	8 / 45 / 0			0·27 (gain) 20;2
chr6:111,982,627-112,025,479	chr6:111,966,419-112,287,583	CN Gain	p=0·0056	0	6q21	p=0·36	36/5	*FYN*	1 / 2 / 0			0·27 (gain) 20;2
chr7:110,980,570-111,558,850	chr7:107,645,325-117,911,160	CN Gain	p<0·0001	71·73	7q31·1 – 7q31·31	p=0·0045	63/7	*MET, WNT2*	49 / 278 / 0	Yes (Grp4)		0·00046 (gain) 50;3
chr7:26,562,652-26,640,208	chr7:25,587,865-26,770,675	CN Gain	p<0·0001	2·43	7p15·2	p=0·00022	60/5	*SKAP2*	10 / 45 / 1			0·00046 (gain) 50;3
chr7:67,357,896-69,126,884	chr7:64,317,306-72,107,612	CN Gain	p=0·0015	3·33	7q11·21 – 7q11·22	p=0·13	63/9	*KCTD7*	45 / 323 / 0			0·00046 (gain) 50;3
chr9:135,461,926-135,535,206	chr9:134,783,624-135,535,206	CN Gain	p=0·0095	0	9q34·13	p=0·24	27/3	*DDX31*	7 / 13 / 0		ns in Grp4 only	0·74 (gain) 13;2
chr9:14,410,492-14,459,033	chr9:14,274,679-15,194,758	CN Gain	p=0·025	0	9p22·3	p=0·94	38/7	*FREM1*	6 / 20 / 0			0·74 (gain) 13;2
chr12:119,434,704-119,567,180	chr12:119,402,945-119,814,605	CN Gain	p=0·00042	23·69	12q24·23	p=0·14	51/7	*HSPB8*	5 / 11 / 0			0·63 (gain) 19;3
chr12:4,377,940-4,425,953	chr12:4,364,943-4,608,984	CN Gain	p=0·0032	30·58	12p13·32	p=0·014	45/4	*CCND2, FGF6*	6 / 8 / 0	Yes (Grp4; 12p13·33 in Grp3)	right next to KCNA1	0·63 (gain) 19;3
chr14:57,149,548-57,453,415	chr14:57,125,694-57,554,180	CN Gain	p<0·0001	10·48	14q22·3	p=0·97	54/10	*OTX2*	2 / 8 / 0	Yes (14q23·1 in Grp3 + Grp4)		0·76 (gain) 18;3
chr17:70,969,175-71,185,781	chr17:56,742,891-81,195,210	CN Gain	p<0·0001	0	17q22 – 17q25·3	p=0·99	86/16	*SOX9, CDK3, AXIN2*	422 / 778 / 15			0·56 (gain) 15;2
chr17:21,713,278-21,976,041	chr17:21,697,647-22,193,626	CN Gain	p<0·0001	100	17p11·2	p=0·19	75/12	*UBBP4*	4 / 45 / 0			0·56 (gain) 15;2
chr18:42,360,772-42,621,429	chr18:42,244,176-42,628,853	CN Gain	p<0·0001	0·30	18q12·3	p=0·033	42/4	*SETBP1*	2 / 3 / 1			0·18 (gain) 28;3
chr18:3,734,349-4,496,774	chr18:3,591,970-4,510,455	CN Gain	p<0·0001	51·08	18p11·31	p=0·67	36/6	*DLGAP1*	7 / 36 / 0			0·18 (gain) 28;3
chr21:17,428,098-17,961,421	chr21:17,407,047-18,308,431	CN Gain	p=0·0071	35·82	21q21·1	p=0·36	36/5	*SNORD74B*	5 / 10 / 0			0·24 (gain) 14;1
chr22:24,343,908-24,358,923	chr22:24,343,908-24,386,848	CN Gain	p=0·0027	100	22q11·23	p=0·060	29/2	*GSTT1*	5 / 41 / 0			0·25 (gain) 6;0
chr1:148,794,708-149,231,196	chr1:148,794,708-149,293,460	CN Loss	p=0·00019	100	1q21·2	p=0·10	19/1	*NBPF25P*	11 / 165 / 0		ns in Grp4 only	0·25 (loss) 6;0
chr3:162,507,077-162,611,127	chr3:162,507,077-162,629,883	CN Loss	p=0·028	100	3q26·1	p=0·15	17/1	-	0 / 70 / 0			0·13 (loss) 10;0
chr8:39,264,684-39,390,007	chr8:39,231,745-39,390,007	CN Loss	p<0·0001	100	8p11·22	p=0·0014	48/3	*ADAM5*	3 / 56 / 0	Yes (8p12 in Grp3 + Grp4)		0·0014 (loss) 31;0
chr8:46,950,145-46,997,244	chr8:46,950,145-48,086,980	CN Loss	p<0·0001	100	8q11·1	p=0·0021	47/3	*ASNSP1*	2 / 73 / 0			0·0014 (loss) 31;0
chr8:1,324,903-1,498,868	chr8:0-1,836,194	CN Loss	p=0·00069	40·48	8p23·3	p=0·17	40/5	*ERICH1*	16 / 293 / 1			0·0014 (loss) 31;0
chr10:135,390,428-135,534,747	chr10:135,116,642-135,534,747	CN Loss	p<0·0001	92·97	10q26·3	p=0·25	38/5	*MTG1*	15 / 63 / 0			0·089 (loss) 12;0
chr10:39,047,547-39,075,616	chr10:38,775,430-39,075,616	CN Loss	p=0·033	100	10p11·1	p=0·27	26/3	*ACTR3BP5*	1 / 44 / 0		ns in Grp4 only	0·089 (loss) 12;0
chr11:2,296,258-2,854,385	chr11:0-3,607,336	CN Loss	p<0·0001	53·75	11p15·5 – 11p15·4	p=0·00079	54/4	*DUSP8, IGF2*	121 / 298 / 4	Yes (Grp3)		0·0072 (loss) 31;1
chr11:55,369,732-55,461,463	chr11:54,835,203-56,913,237	CN Loss	p<0·0001	100	11q11 – 11q12·1	p=0·0043	44/3	many ORs (olf. rec.)	55 / 265 / 0			0·0072 (loss) 31;1
chr13:19,084,823-19,310,900	chr13:19,084,823-20,160,231	CN Loss	p=0·020	100	13q11 – 13q12·11	p=0·016	34/2	*TPTE2*	9 / 102 / 0	Yes (13q12·2 in Grp3)		0·21 (loss) 21;2
chr14:106,534,342-106,566,869	chr14:106,534,342-106,784,498	CN Loss	p=0·0012	100	14q32·33	p=0·75	13/2	*LINC00226*	1 / 140 / 0			0·42 (loss) 3;0
chr15:20,161,372-20,212,798	chr15:20,161,372-20,212,798	CN Loss	p=0·011	100	15q11·1	p=0·88	35/6	-	0 / 41 / 0			0·65 (loss) 1;0
chr16:85,867,977-86,086,370	chr16:85,811,602-87,254,223	CN Loss	p<0·0001	4·47	16q24·1 – 16q24·2	p=0·31	36/5	*FOXF1*	20 / 49 / 0	Yes (Grp3)		0·18 (loss) 8;0
chr16:34,868,574-34,892,930	chr16:34,787,960-35,111,585	CN Loss	p=0·00094	100	16p11·1	p=0·93	39/7	*LINC02167*	1 / 13 / 0			0·18 (loss) 8;0
chr17:3,388,768-3,428,268	chr17:0-5,873,267	CN Loss	p<0·0001	0·67	17p13·3 – 17p13·2	p=0·89	61/11	(not *TP53*)	148 / 460 / 5	Yes (Grp3 + Grp4; 17p13·3)		-
chr22:24,389,278-24,392,203	chr22:24,386,848-24,748,457	CN Loss	p=0·0022	100	22q11·23	p=0·62	14/2	*GSTTP2*	7 / 39 / 0			0·71 (loss) 7;1

GISTIC regions with significant p values from log-rank tests, but no significant survival difference from the respective WCAs are shown with a red background (see matching arrows in appendix p 11 (suppl. figure 5E). Log-rank p values shown in red are significant for both the GISTIC regions and its respective WCA (all cases with gains/losses in the GISTIC region *vs* all cases without gains/losses). Abbreviations: CN, copy number; CNV, copy number variation; WCA, whole chromosomal aberration; ns, not significant.

Supplementary figure 6: SR HIT-SIOP PNET 4 non-WNT/non-SHH-MB ploidy analysis

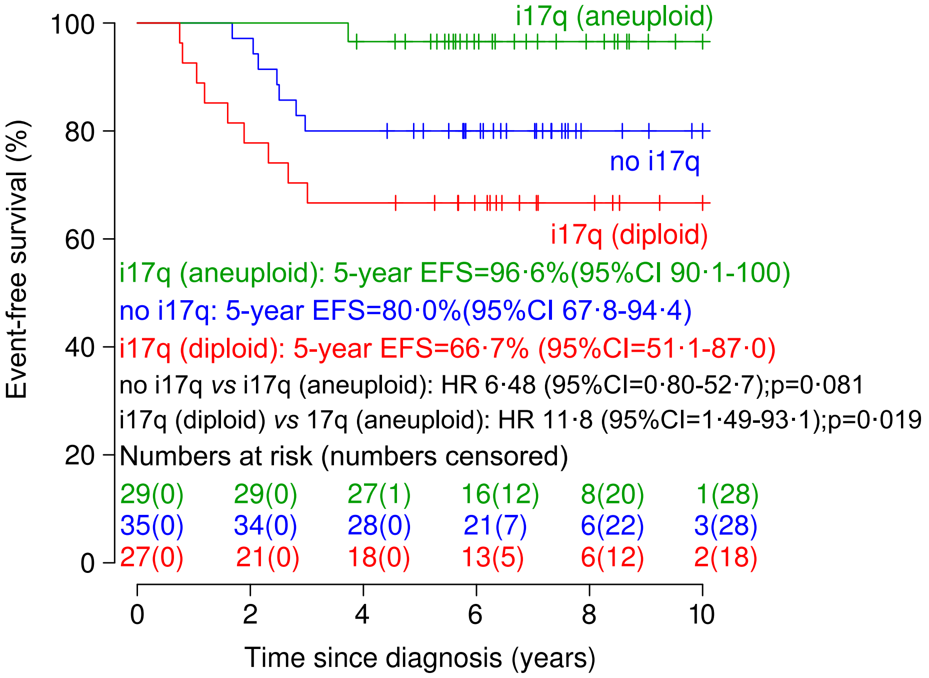

Kaplan-Meier EFS plot for patients with i17q against a diploid background (0–1 chromosomes polysomic), i17q against an aneuploid background (≥2 chromosomes polysomic), and without i17q (independent of ploidy status). Abbreviations: i17q, isochromosome 17q.

Supplementary table 3: AUC, sensitivity and specificity at 5 years for selected stratification schemes.

**SR-MB Grps 3,4**				
**Stratification scheme**	**AUC**	**95%CI**	**Sensitivity (SE)**	**Specificity (SE)**
**0-1 *vs* 2-3 of chrs 7,8,11**	0·765	0·705-0·824	100 (NA)	52·9 (6·1)
**0 chr losses**	0·738	0·654-0·846	88·2 (7·9)	61·8 (5·9)
**WCA cytogenetic**	0·721	0·605-0·836	76·5 (10·3)	67·8 (5·7)
**PNET5 (STR plus *MYCN*)**	0·564	0·464-0·664	18·8 (9·8)	94·0 (2·9)

**SR-MB Group4 Only**				
**Stratification scheme**	**AUC**	**95%CI**	**Sensitivity (SE)**	**Specificity (SE)**
**0-1 *vs* 2-3 of chrs 7,8,11**	0·781	0·716-0·846	100 (NA)	56·1 (6·6)
**0 chr losses**	0·753	0·642-0·865	85·7 (9·4)	64·9 (6·4)
**WCA cytogenetic**	0·655	0·514-0·795	64·3 (12·9)	66·7 (6·3)
**PNET5 (STR plus *MYCN*)**	0·545	0·448-0·642	14·3 (9·4)	94·6 (3·0)

Supplementary figure 7: AUC of (A) cytogenetic stratification and (B) cytogenetic schemes *versus* a published stratification scheme

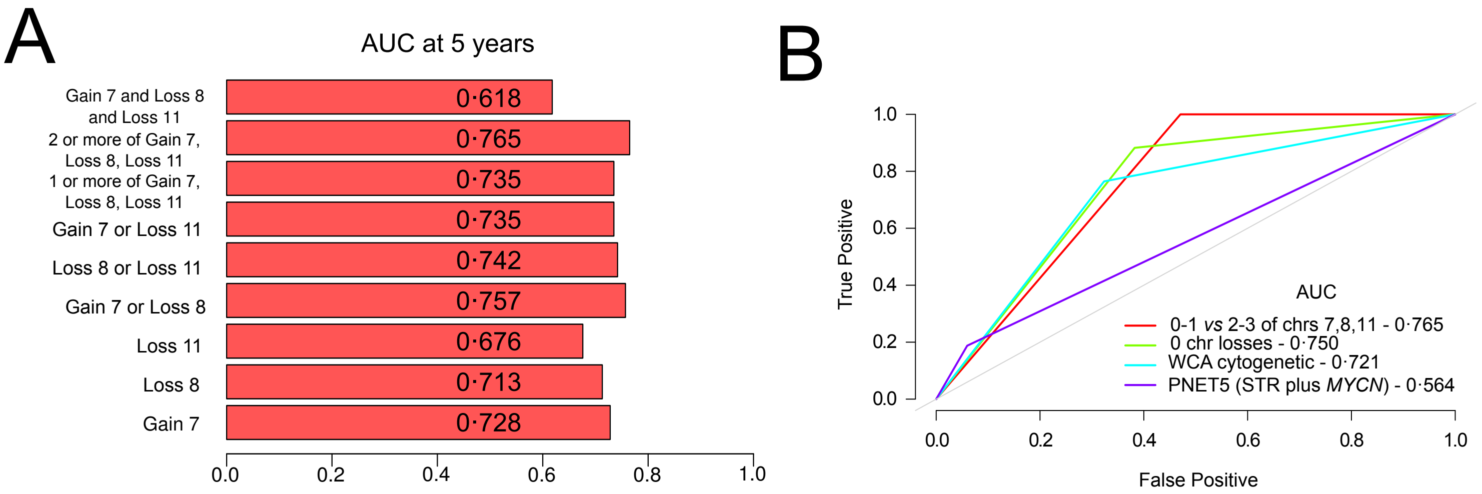

(A) Investigation of AUC at 5 years to investigate optimal combinations of cytogenetic features for predicting event-free survival. (B) Time-dependent ROC curves at 5 years demonstrate performance of three stratification schemes; 0–1 *vs* 2–3 changes of chrs7, 8, 11: see scheme outlined in figure 4C; 0 chr losses: patients stratified into 0 chromosomal losses *vs* 1 or more losses; WCA cytogenetic: biologically determined sample clusters identified in figure 3B. For each, AUC is stated and PNET5 stratification is shown for comparison.

Supplementary figure 8: Prognostic significance of WCA in non-WNT/non-SHH-MB within HIT-SIOP PNET 4

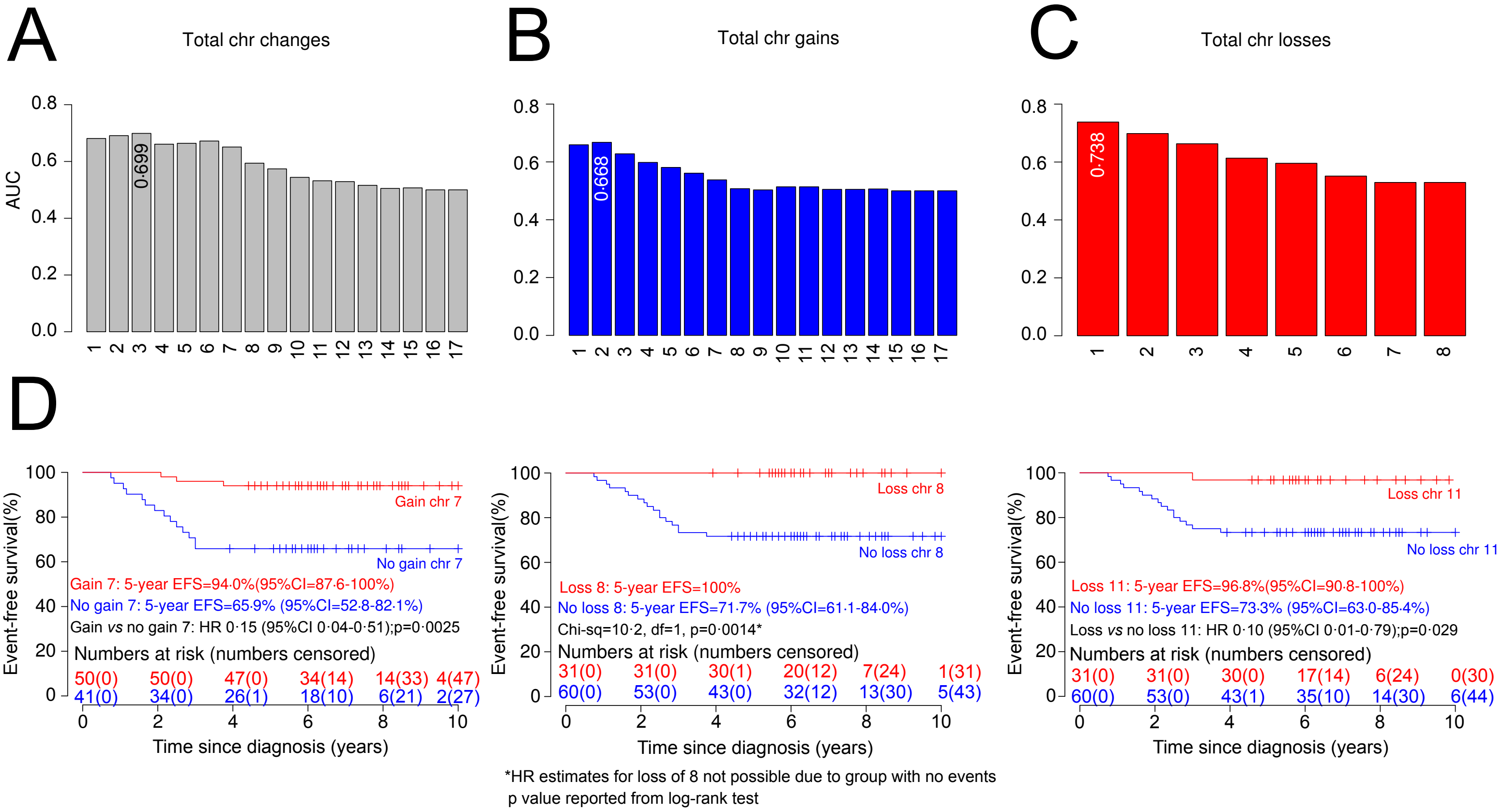

(A–C) Time-dependent AUC at 5 years for varying numbers of total WCA (A), gains (B) and losses (C). Numbers of WCA with maximal AUC are labelled. (D) Kaplan-Meier EFS plots for identified prognostic WCA; chr7 gain, chr8 loss and chr 11 loss.

Supplementary table 4. Univariable Cox proportional hazards models for clinical, molecular and recurrent cytogenetic features.

		Univariable analysis				
		n	HR	95% CI	95% CI	p
Cytogenetic change	Gain 4	28/91	0·46	0·13	1·61	0·23
	Gain 5	16/91	0·61	0·14	2·68	0·52
	Gain 6	20/91	0·44	0·10	1·94	0·28
	Gain 7	50/91	0·15	0·04	0·51	0·0025
	Loss 8*	31/91	NA	NA	NA	0·0014
	Loss 11	31/91	0·10	0·01	0·79	0·029
	Gain 12	19/91	0·74	0·21	2·58	0·64
	Loss 13	21/91	0·40	0·09	1·75	0·22
	Gain 14	18/91	0·82	0·24	2·86	0·76
	Gain 17	15/91	0·65	0·15	2·84	0·57
	Gain 18	28/91	0·44	0·13	1·53	0·20
	Gain 21	14/91	0·32	0·04	2·38	0·26
	i17q	56/91	0·92	0·35	2·41	0·86
Clinical feature	Female sex	30/91	0·24	0·05	1·05	0·06
	Hyperfractionated RTX	48/91	0·46	0·17	1·24	0·13
	Subtotal resection	12/87	0·38	0·05	2·89	0·35
	MYCN amplification	9/91	0·53	0·07	4·03	0·54

*Estimates for loss of 8 not possible due to group with no events
p value reported from log-rank test

Univariable Cox models of clinical, molecular and recurrent (frequency >15%) cytogenetic features. For each variable, frequencies, hazard ratios, 95% CIs and p value from Wald tests are shown. Features significantly associated with event-free survival are shown in red. Hyperfractionated RTX - hyperfractionated radiotherapy.

Supplementary table 5: Analysis of all individual whole chromosomal aberrations for SR-Group4 and non-WNT/non-SHH-MB

Chromosome	WCA	Grp4 (n=76; 14 events)		non-WNT/non-SHH (n=91; 17)		Chromosome
		n/events	p value (log-rank)	n/events	p value (log-rank)	
Chr1	Loss<br>Gain	4/0<br>8/1	0·35<br>0·65	6/0<br>11/1	0·25<br>0·39	Chr1
Chr2	Loss<br>Gain	2/0<br>9/2	0·52<br>0·75	3/0<br>9/2	0·42<br>0·79	Chr2
Chr3	Loss<br>Gain	8/0<br>1/0	0·18<br>0·65	10/0<br>2/0	0·13<br>0·52	Chr3
Chr4	Loss<br>Gain	1/0<br>21/3	0·650<br>0·610	1/0<br>28/3	0·65<br>0·22	Chr4
Chr5	Loss<br>Gain	3/0<br>12/2	0·43<br>0·90	4/0<br>16/2	0·35<br>0·51	Chr5
Chr6	Loss<br>Gain	1/0<br>17/2	0·65<br>0·43	1/0<br>20/2	0·65<br>0·27	Chr6
Chr7	Gain	41/3	0·0059	50/3	0·00046	Chr7
Chr8	Loss<br>Gain	30/0<br>0/0	0·0011<br>-	31/0<br>2/0	0·0014<br>0·52	Chr8
Chr9	Loss<br>Gain	0/0<br>11/2	-<br>0·99	2/0<br>13/2	0·52<br>0·74	Chr9
Chr10	Loss<br>Gain	9/0<br>1/0	0·15<br>0·65	12/0<br>1/0	0·089<br>0·65	Chr10
Chr11	Loss	26/1	0·019	31/1	0·0072	Chr11
Chr12	Gain	14/3	0·86	19/3	0·633	Chr12
Chr13 (q-arm only)	Loss<br>Gain	21/2<br>4/0	0·20<br>0·35	21/2<br>8/0	0·21<br>0·18	Chr13 (q-arm only)
Chr14 (q-arm only)	Loss<br>Gain	3/0<br>14/3	0·43<br>0·80	3/0<br>18/3	0·42<br>0·76	Chr14 (q-arm only)
Chr15 (q-arm only)	Loss<br>Gain	1/0<br>7/2	0·65<br>0·51	1/0<br>11/2	0·65<br>0·91	Chr15 (q-arm only)
Chr16	Loss<br>Gain	6/0<br>4/2	0·25<br>0·068	8/0<br>5/2	0·18<br>0·20	Chr16
Chr17	Gain	12/2	0·87	15/2	0·56	Chr17
Chr18	Gain	23/3	0·41	28/3	0·18	Chr18
Chr19	Loss<br>Gain	3/0<br>5/1	0·43<br>0·90	3/0<br>6/1	0·42<br>0·91	Chr19
Chr20	Loss<br>Gain	8/0<br>5/1	0·18<br>0·90	8/0<br>7/1	0·18<br>0·77	Chr20
Chr21 (q-arm only)	Loss<br>Gain	10/1<br>10/1	0·44<br>0·47	10/1<br>14/1	0·44<br>0·24	Chr21 (q-arm only)
Chr22 (q-arm only)	Loss<br>Gain	4/1<br>6/0	0·81<br>0·25	7/1<br>6/0	0·71<br>0·25	Chr22 (q-arm only)

Supplementary figure 9: Chromosomal aberrations comparison barplots between HIT-SIOP PNET 4 SR-MB and validation cohorts

A

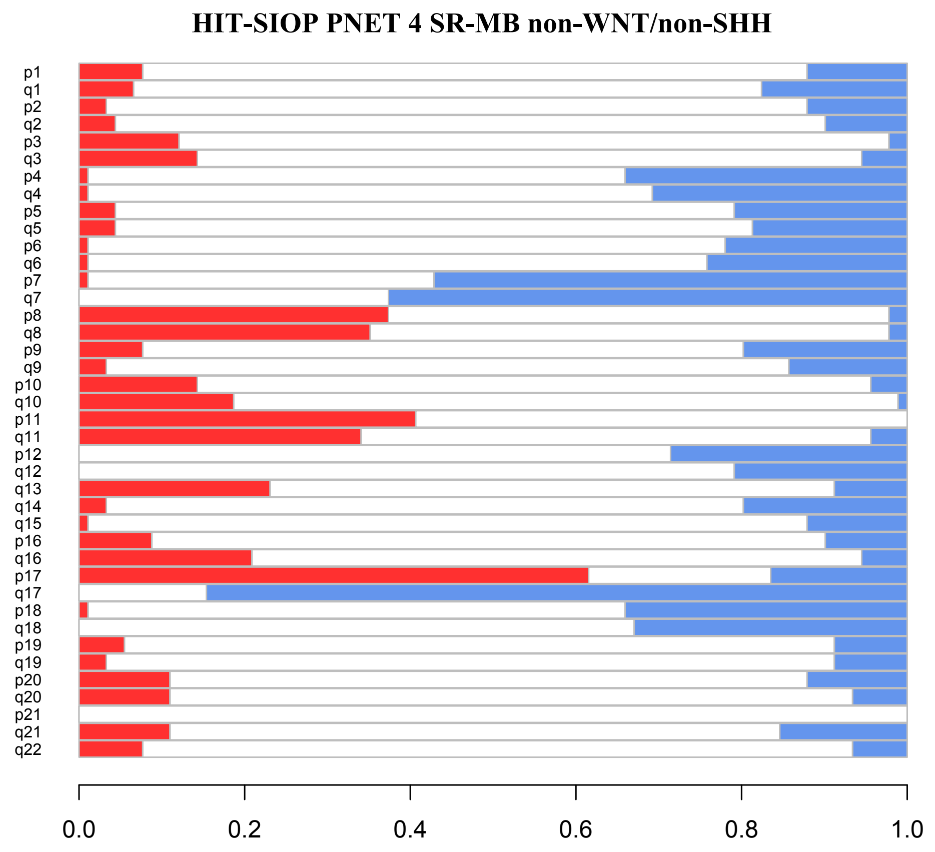

B

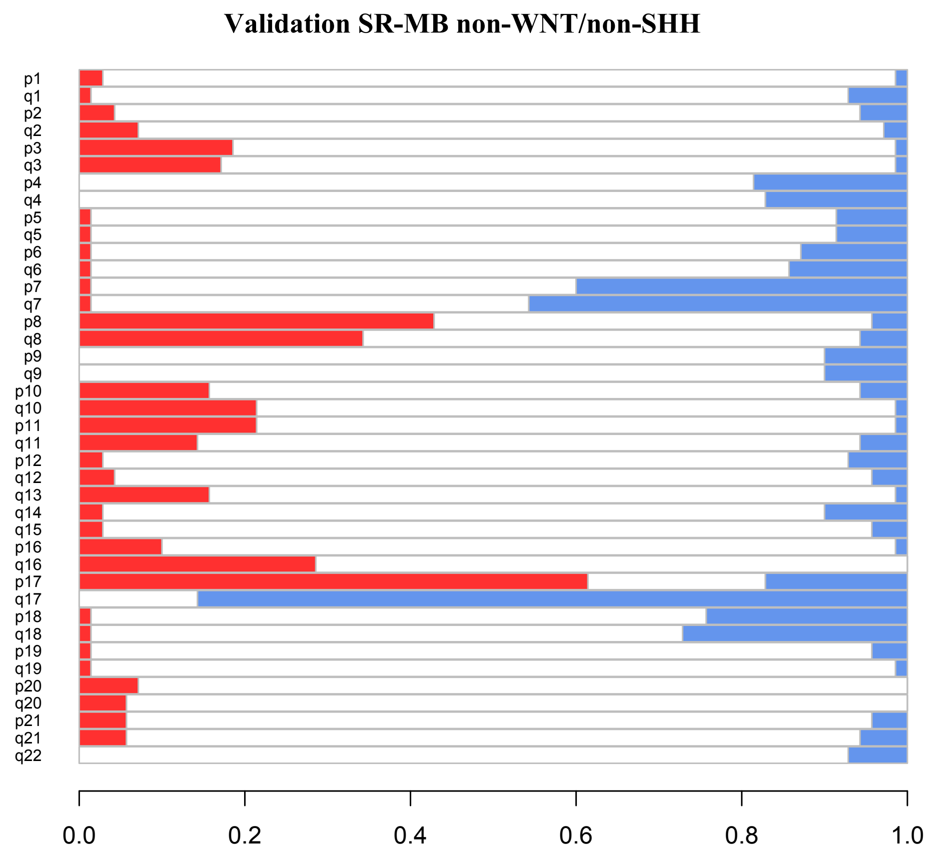

(A–B) Barplots show frequencies of p- and q-arm gains (blue) and losses (red) for all chromosomes from HIT-SIOP PNET4 non-WNT/non-SHH SR-MB assessed by MIP array (n=91; A) and the validation cohort assessed by 450k methylation array (n=70; B).

Supplementary figure 10: Performance of cytogenetic stratification schemes in validation cohort

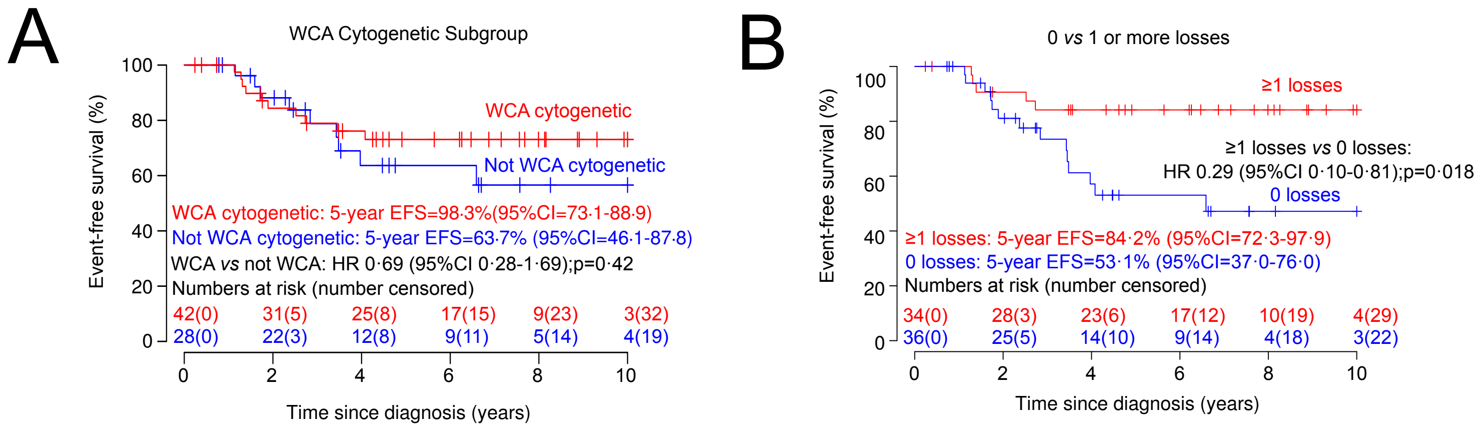

Kaplan-Meier EFS plot for (A) validation cohort patients stratified by WCA cytogenetic subgroup and (B) by 0 *vs* 1 or more whole chromosomal losses

Supplementary table 6: Relationship between Group3/4 subtypes described by Schwalbe et al. and cytogenetic risk scheme

	Favourable-risk	High-risk	
$MB_{Grp4\ High\text{-}risk}$	0	29	
$MB_{Grp4\ Low\text{-}risk}$	19	11	
$MB_{Grp3\ Low\text{-}risk}$	1	1	
$MB_{Grp3\ High\text{-}risk}$	0	9	p<0·0001

Cross-tabulation of WCA-defined risk groups and Group3/4 disease subtypes described by Schwalbe et al.[3] in SR-MB validation cohort.

Supplementary table 7: Relationship between Group3/4 subtypes described by Northcott et al. and cytogenetic risk scheme

	Favourable-risk	High-risk	
I	0	3	
II	0	5	
III	0	2	
IV	0	0	
V	1	3	
VI	11	1	
VII	8	7	
VIII	0	29	p<0·0001

Cross-tabulation of WCA-defined risk groups and Group3/4 disease subtypes described by Northcott et al.[12] in SR-MB validation cohort.

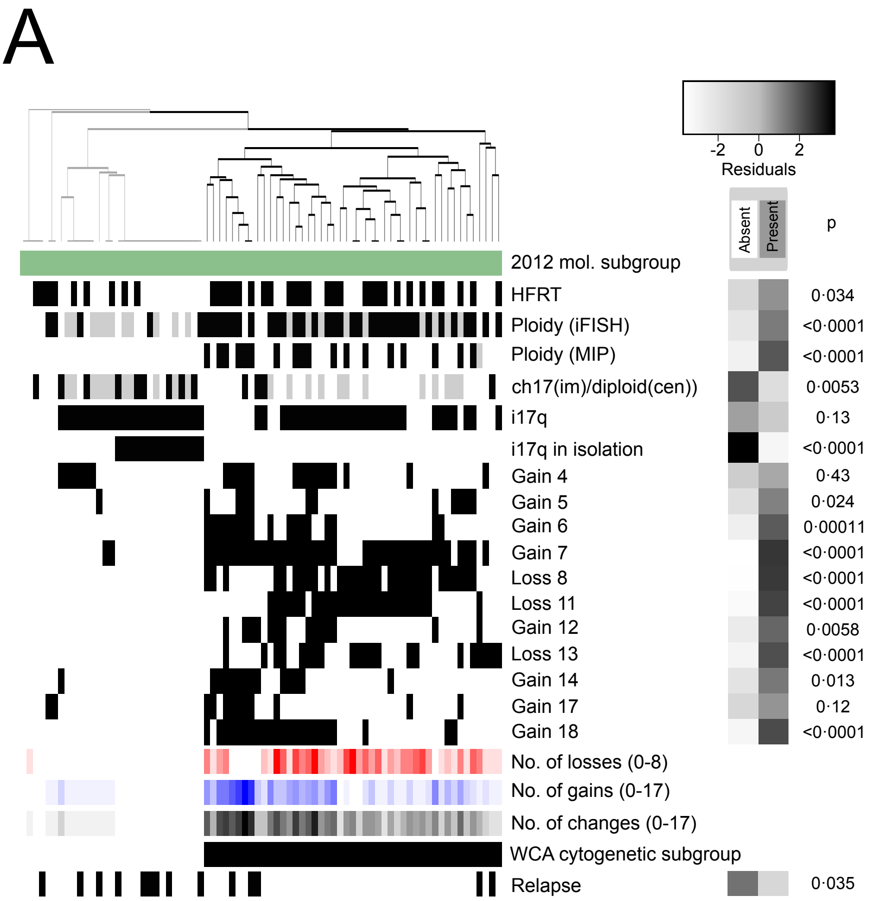

B

		Univariate					
		n	HR	95% CI	95% CI	p
Cytogenetic change	Gain 4	21/76	0·72	0·20	2·58	0·61
	Gain 5	12/76	0·91	0·20	4·07	0·90
	Gain 6	17/76	0·56	0·12	2·48	0·44
	Gain 7	41/76	0·20	0·06	0·71	0·013
	Loss 8*	30/76	NA	NA	NA	0·0011
	Loss 11	26/76	0·13	0·02	0·98	0·048
	Gain 12	14/76	1·13	0·32	4·05	0·85
	Loss 13	21/76	0·39	0·09	1·75	0·22
	Gain 14	14/76	1·18	0·33	4·24	0·80
	Gain 17	12/76	0·88	0·20	3·95	0·87
	Gain 18	23/76	0·59	0·16	2·10	0·41
	i17q	52/76	0·85	0·28	2·54	0·77
Clinical feature	Female sex	27/76	0·27	0·06	1·20	0·086
	HFRT	36/76	0·27	0·08	0·98	0·047
	STR	11/74	0·43	0·06	3·29	0·42
	MYCN amplification	9/76	0·54	0·07	4·16	0·56

*Estimates for loss of 8 not possible due to group with no events.
p values reported from log-rank test

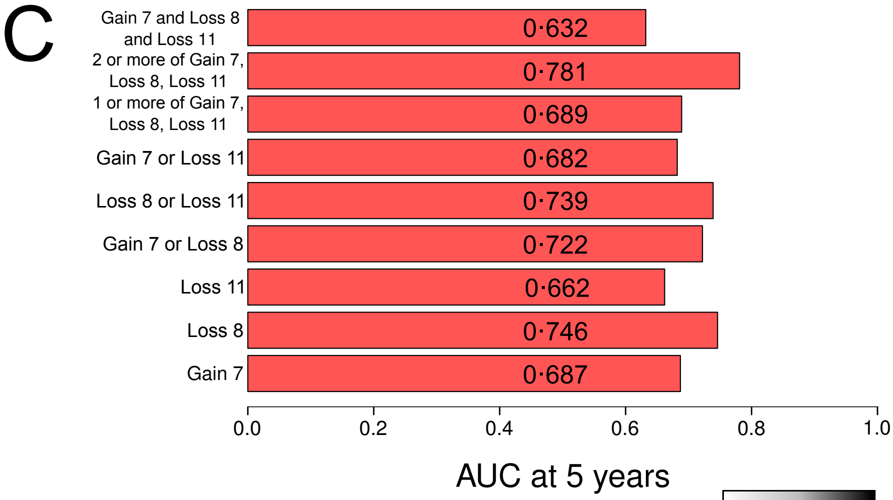

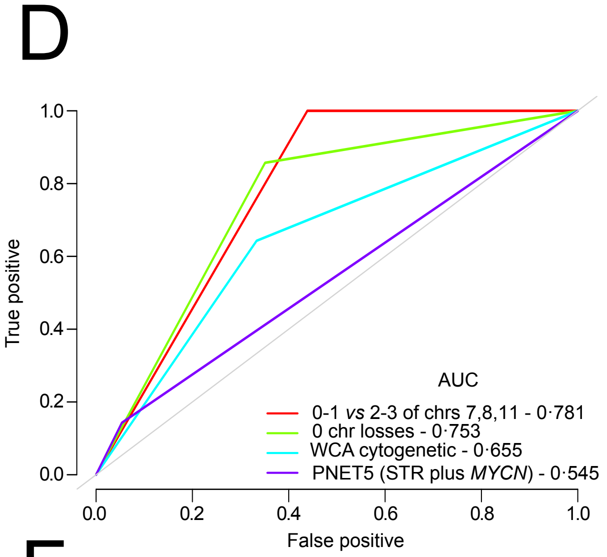

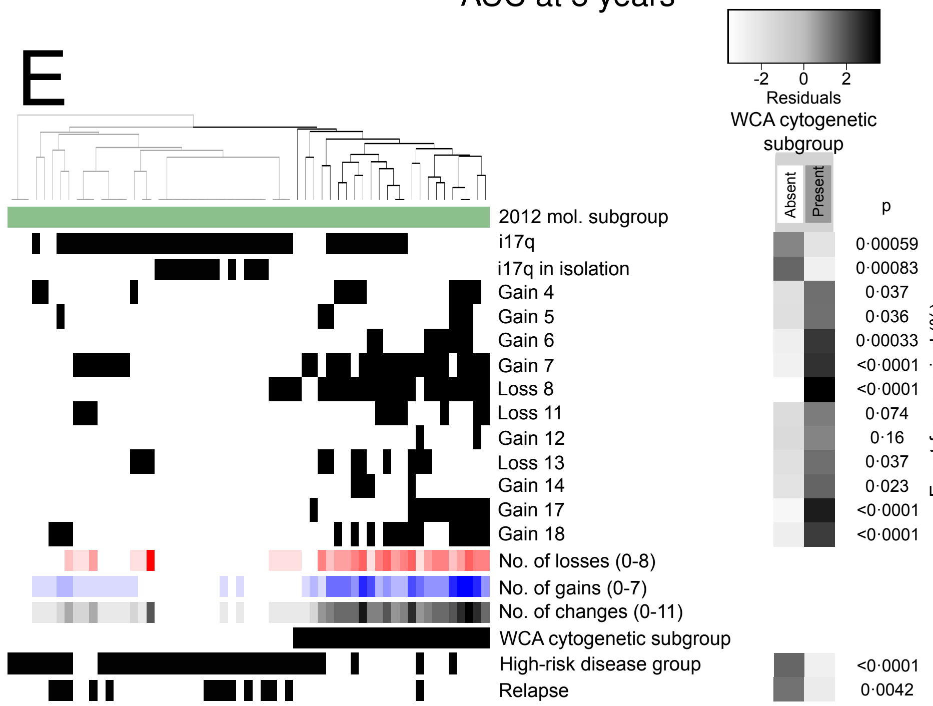

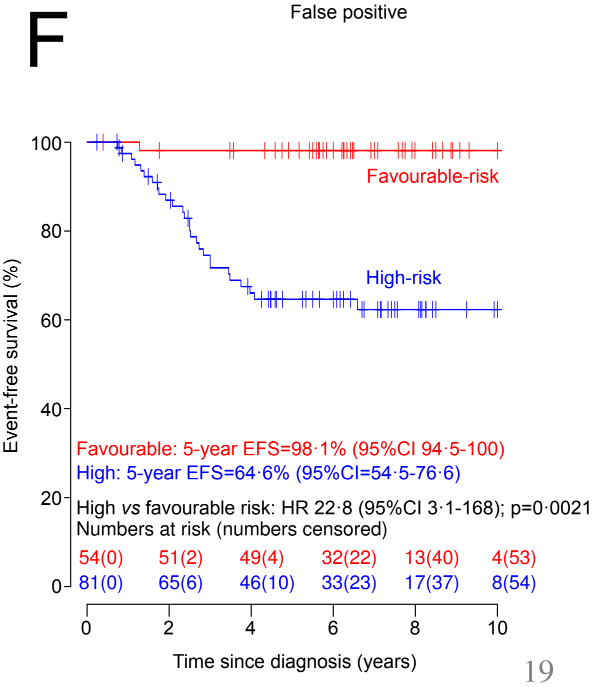

Supplementary figure 11: Analysis of Group4 SR-MB identifies the same molecularly defined variants which can be stratified into favourable and high-risk groups

(A) Unsupervised clustering of chromosomal features identifies distinct, clinically relevant WCA cytogenetic subgroups. HFRT, ploidy measured by iFISH, ploidy measured by MIP, chr17 defects measured by iFISH and specific cytogenetic defects is shown black. Missing data are shaded grey. Residuals from chi-squared tests indicate where WCA cytogenetic group enrichment has occurred (darker shades of grey indicate stronger relationships), p values are from Fisher's exact tests. Scale bars for residuals are shown. The total numbers of whole chromosomal losses, gains and changes are shown in colour scales shaded red, blue and black respectively. Increasing intensity indicates a larger number of changes. Chromosomal changes with incidence >15% are shown.

(B) Univariable Cox models of clinical and recurrent (frequency >15%) cytogenetic features. For each variable, frequencies, hazard ratios, 95% CIs and p value from Wald tests are shown. Features significantly associated with survival are shown in red. (C) Investigation of AUC at 5 years to investigate optimal combinations of cytogenetic features for predicting survival. (D) Time-dependent ROC curves at 5 years demonstrate performance of three simple risk-stratification schemes. 0–1 *vs* 2–3 changes of chrs 7,8,11: see scheme outlined in figure 4D; 0 chr losses: patients stratified into 0 chromosomal losses *vs* 1 or more losses; WCA cytogenetic: biologically determined sample clusters identified in part A. For each, AUC is stated and PNET 5 stratification is shown for comparison. (E) Unsupervised clustering of chromosomal features in independent SR-MB Group4 cohort validated distinct, clinically relevant WCA cytogenetic subgroups. (F) Kaplan-Meier plot for combined molecularly characterised HIT-SIOP PNET4 and validation cohorts, stratified by derived WCA-defined risk scheme (favourable-risk – positive for ≥2 changes from chr7 gain, chrs8/11 loss; high-risk – fewer than 2 changes).

References

1. Lannering B, Rutkowski S, Doz F, et al. Hyperfractionated versus conventional radiotherapy followed by chemotherapy in standard-risk medulloblastoma: results from the randomized multicenter HIT-SIOP PNET 4 trial. *J Clin Oncol* 2012; **30**: 3187–93.
2. Thompson EM, Hielscher T, Bouffet E, et al. Prognostic value of medulloblastoma extent of resection after accounting for molecular subgroup: a retrospective integrated clinical and molecular analysis. *Lancet Oncol* 2016; **17**: 484–95.
3. Schwalbe EC, Lindsey JC, Nakjang S, et al. Novel molecular subgroups for clinical classification and outcome prediction in childhood medulloblastoma: a cohort study. *Lancet Oncol* 2017; **18**: 958–971.
4. Shih DJH, Northcott PA, Remke M, et al. Cytogenetic prognostication within medulloblastoma subgroups. *J Clin Oncol* 2014; **32:** 886–96.
5. Clifford SC, Lannering B, Schwalbe EC, et al. Biomarker-driven stratification of disease-risk in non-metastatic medulloblastoma: Results from the multi-center HIT-SIOP PNET4 clinical trial. *Oncotarget* 2015; **6**: 38827–39.
6. Chang CH, Housepian EM, Herbert C Jr. An operative staging system and a megavoltage radiotherapeutic technic for cerebellar medulloblastomas. *Radiology* 1969; **93**: 1351-9.
7. Hovestadt V, Remke M, Kool M, et al. Robust molecular subgrouping and copy-number profiling of medulloblastoma from small amounts of archival tumour material using high-density DNA methylation arrays. *Acta Neuropathol* 2013; **125**: 913–16.
8. Northcott PA, Shih DJ, Remke M, et al. Rapid, reliable, and reproducible molecular sub-grouping of clinical medulloblastoma samples. *Acta Neuropathol* 2012; **123**: 615–26.
9. Schwalbe EC, Hicks D, Rafiee G, et al. Minimal methylation classifier (MIMIC): A novel method for derivation and rapid diagnostic detection of disease-associated DNA methylation signatures. *Sci Rep* 2017; **7**: 13421.
10. Wang Y, Cottman M, Schiffman JD. Molecular inversion probes: a novel microarray technology and its application in cancer research. *Cancer Genet* 2012; **205**: 341–55.
11. Beroukhim R, Getz G, Nghiemphu L, et al. Assessing the significance of chromosomal aberrations in cancer: methodology and application to glioma. *Proc Natl Acad Sci U S A* 2007; **104**: 20007–12.
12. Northcott PA, Buchhalter I, Morrissy AS, et al. The whole-genome landscape of medulloblastoma subtypes. *Nature* 2017; **547**: 311–317.
13. Northcott PA, Shih DJ, Peacock J, et al. Subgroup-specific structural variation across 1,000 medulloblastoma genomes. *Nature* 2012; **488**: 49–56.
